# Recombinase-Mediated Reprogramming and Dystrophin Gene Addition in *mdx* Mouse Induced Pluripotent Stem Cells

**DOI:** 10.1371/journal.pone.0096279

**Published:** 2014-04-29

**Authors:** Chunli Zhao, Alfonso P. Farruggio, Christopher R. R. Bjornson, Christopher L. Chavez, Jonathan M. Geisinger, Tawny L. Neal, Marisa Karow, Michele P. Calos

**Affiliations:** Department of Genetics, Stanford University School of Medicine, Stanford, California, United States of America; University of Minnesota Medical School, United States of America

## Abstract

A cell therapy strategy utilizing genetically-corrected induced pluripotent stem cells (iPSC) may be an attractive approach for genetic disorders such as muscular dystrophies. Methods for genetic engineering of iPSC that emphasize precision and minimize random integration would be beneficial. We demonstrate here an approach in the *mdx* mouse model of Duchenne muscular dystrophy that focuses on the use of site-specific recombinases to achieve genetic engineering. We employed non-viral, plasmid-mediated methods to reprogram *mdx* fibroblasts, using phiC31 integrase to insert a single copy of the reprogramming genes at a safe location in the genome. We next used Bxb1 integrase to add the therapeutic full-length dystrophin cDNA to the iPSC in a site-specific manner. Unwanted DNA sequences, including the reprogramming genes, were then precisely deleted with Cre resolvase. Pluripotency of the iPSC was analyzed before and after gene addition, and ability of the genetically corrected iPSC to differentiate into myogenic precursors was evaluated by morphology, immunohistochemistry, qRT-PCR, FACS analysis, and intramuscular engraftment. These data demonstrate a non-viral, reprogramming-plus-gene addition genetic engineering strategy utilizing site-specific recombinases that can be applied easily to mouse cells. This work introduces a significant level of precision in the genetic engineering of iPSC that can be built upon in future studies.

## Introduction

One of the most exciting applications of our growing knowledge of stem cells is the potential to use them in cell therapy strategies for degenerative disorders. In considering which type of stem cells to employ in such therapies, pluripotent stem cells, including embryonic stem cells (ESC) and induced pluripotent stem cells (iPSC) [1], [2] are appealing, because they have an unlimited lifespan. This feature would allow the cellular expansion needed to carry out genetic engineering methods to repair causative mutations, as well as permitting generation of the large numbers of cells needed to repair an extensive tissue target. iPSC have the additional attraction of being derived from patients, which may alleviate immunological rejection of transplanted cells [3], [4].

Muscular dystrophies represent attractive potential targets for stem cell therapy approaches, since muscle tissue is accessible and engraftable [5]. Many forms of muscular dystrophy exist, resulting from mutation of various genes that affect muscle cells [6]. Among these disorders, Duchenne muscular dystrophy (DMD) is a severe genetic disease resulting from mutation of the X-linked dystrophin gene [7]. In the absence of dystrophin, muscle fibers progressively break down, producing muscle weakness that typically leads to wheelchair use by the teens and respiratory or cardiac failure in the twenties. DMD affects 1 in 3500 males and is currently incurable [8]. While a variety of gene therapy and pharmacological approaches are being developed [9], the degenerative nature of muscular dystrophies makes a cell therapy approach attractive, because it has the potential to replace the muscle fibers that are lost during progression of these disorders [5].

In recent years, several studies have demonstrated the ability of ESC and iPSC to differentiate into engraftable muscle precursors [10]–[20]. This ability is a key attribute for feasibility of the pluripotent stem cell approach. Additionally, if patient-derived iPSC are used in a therapeutic strategy for DMD, the endogenous mutation in the dystrophin gene must be repaired or compensated for, such that the cells express functional dystrophin. An impediment to repair of dystrophin is the large size of the gene and protein, since even the cDNA is ∼14 kb in length [7]. Furthermore, the genetic engineering methods employed to produce cellular reprogramming and to provide for repair of dystrophin should be as safe and minimally disruptive to the host genome as possible.

In the iPSC studies addressing DMD to date, retroviruses have been used to create the iPSC [13], [15]–[19]. This reprogramming method typically produces multi-copy, random integration of vectors into the genome, which can lead to tumorigenesis and other abnormalities [1], [2]. In most of these studies, iPSC from wild-type individuals were used [13], [15], [19], which does not model the immune tolerance advantage that would accompany the repair of patient-derived cells. By contrast, one study involved introducing wild-type *DYSTROPHIN* on a supernumerary human artificial chromosome vector [18], a strategy with unknown safety implications. In another study, the repair strategy involved compensation for the dystrophin deficiency by random insertion into the genome of a Sleeping Beauty transposon vector carrying a truncated version of the utrophin coding sequence [16]. This procedure typically produces multicopy, random integration. In addition, some iPSC strategies have employed random integration of lentiviral vectors to introduce genes to enhance myogenic differentiation and/or tracking genes. The final results were iPSC carrying multiple, uncharacterized random integration events.

To move away from random integration and toward strategies featuring controlled genomic modification, we set out to create a non-viral gene therapy/cell therapy strategy that employed genetic engineering methods that had a greater degree of precision. We used site-specific recombinases to control the location and copy number of genetic manipulations. To this end, phiC31 integrase was used to mediate initial placement of a single copy of a reprogramming plasmid into the genome at a safe location. A second phage integrase, Bxb1, was used to place the full-length dystrophin coding sequence into the same location, and Cre resolvase was utilized to excise unwanted sequences. We pursued differentiation and engraftment procedures to illustrate that iPSC genetically engineered cells in a defined fashion retained the ability to differentiate and engraft. This study represents a step toward the development of a well-defined genetic engineering strategy that can be applied to iPSC for the development of cell therapies.

## Materials and Methods

### Ethics statement

The Stanford Administrative Panel on Laboratory Animal Care approved all procedures performed on animals in protocol number 15766, assurance number A3213-01. The Stanford Comparative Medicine program is accredited by the Association for Accreditation and Assessment of Laboratory Animal Care International.

### Cell culture

Mouse ESC were from Applied Stem Cell (Menlo Park, CA). Mouse adult fibroblasts were isolated from *mdx* mice (Jackson Lab strain C57BL/10ScSn-*Dmd^mdx^*/J, stock number 001801). All murine iPSC lines used were generated in this study; no human cells were used. ESC and iPSC were cultured in DMEM, plus 20% FBS and 1000 U/ml LIF (leukaemia inhibitory factor; Chemicon, Billerica, MA), 2 mM glutamine (Gibco, Grand Island, NY), 1 mM sodium pyruvate (Gibco), 0.1 mM β-mercaptoethanol (Gibco), and 2 mM non-essential amino acids (NEAA, Gibco). Medium was changed every day until confluent. 0.05% trypsin-EDTA (Gibco) was used to passage cells.

### Plasmids

Details of the construction of pCOBLW and pKHLB-mDystr are provided in the Supplementary Materials and Methods (File S1). Plasmid pCAG-Cre [21], expressing Cre resolvase, was purchased from Addgene (#13775).

### Co-nucleofection of pCOBLW and pVI into *mdx* fibroblasts

For each nucleofection reaction, 2×10^6^ adult fibroblasts (AF) were nucleofected (Lonza, Walkersville, MD) according to the manufacturer's instructions using the MEF nucleofector kit I (program T-20 on the Amaxa Nucleofector II). A total of 3 µg of DNA were used per nucleofection, with varying ratios by mass of 1∶1, 1∶7, 1∶10, 1∶20 of pCOBLW and pVI [22]. Fibroblasts were harvested for nucleofection at 80% confluency. 48 hours post-nucleofection, the cells were transferred onto irradiated CF1 feeder cells plated on 10 cm dishes coated with 0.1% gelatin at least 30 min in advance. Culture medium was switched to ESC culture medium, and medium was changed every day until colonies were picked. Colonies became visible starting from day 8–10 and were picked on day 20–26.

### Southern blotting

Genomic DNA was extracted using the gDNA Midi Kit (Zymo Corp, Irvine, CA). 7–15 µg genomic DNA from each iPSC line were digested overnight with *Eco*32I or *Eco*O109I (Fermentas) and resolved by electrophoresis on 0.8–1.0% agarose gels at 16–22 V for 6–10 h. DNA was transferred using the Whatman kit (Schleicher and Schuell, Keene, NH) and nitrocellulose membrane (Hybond-N; GE Healthcare, Piscataway, NJ). The Turboblotter transfer apparatus (Schleicher and Schuell) was used following the manufacturer's instructions. A DIG-labeled GFP probe generated by the DIG High Prime Labeling and Detection Starter Kit II (Roche, Indianapolis, IN) was then hybridized to the membrane following the manufacturer's instructions.

### Ligation-Mediated PCR (LM-PCR)

1 µg of genomic DNA was digested with *Mse*I (New England Biolabs, Ipswich, MA) in a 10 µl reaction at 37°C for 4 hours before being heat-killed at 65°C for 20 minutes. Linker adapters at concentration 10 µM and possessing a TA overhang were then ligated to the digested ends of 500 ng of the digest using high concentration T4 DNA ligase (NEB). The ligation reaction was incubated at 16°C overnight. Following ligation, polymerase chain reaction (PCR) was performed with 2 µl of the ligation reaction utilizing Phusion High Fidelity Polymerase (NEB) using the following primer pairs: 1) Adaptor P1 (5′-GTAATACGACTCACTATAGG*G*C-3′) and AttBF2 (5′-ATGTAGGTCACGGTCTCGAA*G*C-3′) and 2) Adaptor P1 and AttBR1 (5′-TCCCGTGCTCACCGTGACC*A*C-3′). The asterisk is used to denote a phosphorothioate bond. For these PCR reactions the following thermocycler protocol was used: touchdown PCR with 98°C denaturing time of 15 seconds, 60°C–55°C annealing time of 30 seconds, and 72°C extension time of 30 seconds. A second round of PCR amplification was carried out using the same thermocycler protocol and 2 µl of a 1∶100 dilution of the first round PCR utilizing Phusion and the following sets of primers: 1) Adaptor P2 (5′-AGGGCTCCGCTTAAGGG*A*C-3′) and AttBF3 (5′-CGAAGCCGCGGTG*C*-3′) and 2) Adaptor P2 and AttBR2 (5′-ACTACCGCCACCTCG*A*C-3). Gel electrophoresis of the products was carried out using a 1% agarose gel at 100 V. Bands were excised and cloned into the pJET1.2 blunt cloning vector (CloneJET kit; Thermo-Fisher, Waltham, MA) and transformed into α-Select electrocompetent *E. coli* (Bioline). The pJET forward sequencing primer (Thermo-Fisher) was used for Sanger sequencing.

### Dystrophin addition and Cre excision

iPSC were pre-plated on 0.1% gelatin-coated tissue culture dishes for 45–60 min to ensure a pure population free of feeder cells for nucleofection. 3×10^6^ *mdx* iPS cells were nucleofected (Lonza) per reaction according to the manufacturer's instructions using the Mouse ES Cell Nucleofection Kit, program A-30, Amaxa Nucleofector II, using 12 µg total DNA per nucleofection (pKHLB-mDystr∶pCS-Bxb1, ratio 1∶1 by mass). Cells were then plated on irradiated DR4 feeder cells on 10 cm dishes coated with 0.1% gelatin. After 48 hours, 2 µg/ml puromycin (InvivoGen, San Diego, CA) selection was started for 7–10 days. Puromycin-resistant colonies were picked and expanded. PCR was used to detect correct integration by amplifying Bxb1 *att*R and *att*L junctions within the integrated pCOBLW vector. 100 ng of genomic DNA were used in 25 µl reactions, with Phusion as the polymerase. Primers for the *att*R junction were: 5'- AGCACGACGGCGCTGCCCAGAC*C*C-3', 5'-CCGGAGGCCCGGCATTCTGCAC*G*C-3' and for the *attL* junction: 5′-AGACACAAAGTCCCCAAGGCGG*C*C, 5′- CGCGGGGTTCGGTCCCAGGGCT*G*G-3', where asterisks are used to denote phosphorothioate bonds. The thermocycler program used for Bxb1 *attR* junction detection was: 98°C for 30 sec; 10 cycles of [98°C for 7 sec, 83–78°C for 10 sec (0.5°C/cycle decrements), 72°C for 18 sec]; and 40 cycles of [98°C for 7 sec, 78°C for 10 sec, 72°C for 18 sec]; and a final elongation at 72°C for 1 min. The thermocycler program used for Bxb1 *attL* detection was: 98°C for 30 sec; 10 cycles of [98°C for 7 sec, 77–72°C for 10 sec (0.5°C/cycle decrements), 72°C for 13 sec]; and 40 cycles of [98°C for 7 sec, 78°C for 10 sec, 72°C for 13 sec]; and a final elongation at 72°C for 1 min. The sizes of the *attR* and *attL* products were 591 bp and 431 bp, respectively.

Several subclones with correctly targeted Bxb1 integrations were cultured on irradiated CF1 feeder on 0.1% gelatin-coated plates. Cells were nucleofected as above with pCAG-Cre. 2–4 days post-nucleofection, colonies lacking GFP expression, as judged visually, were picked and expanded. Genomic DNA was isolated for use in nested PCR to detect Cre junctions indicating successful excision. 100 ng of genomic DNA was used in 50 µl reactions with HotStarTaq Plus (Qiagen). The first round of the nested PCR used primers 5'-GCGGGGGTCGTTGGGCGGTCAG*C*C-3' and 5'-CCACCCACCGTGCCCACTGGC*C*A-3'. The second round of the nested PCR used primers 5'-GCGGCCGCTCGAGAAGCTTAAG-3' and 5'-GCCCGACCCTCCCCTGGCACAACG-3'. For the second-round PCR, the primary PCR reaction was diluted 64× in a separate microcentrifuge tube. 6.4 µl of this dilution was used in a 50 µl reaction. The thermocycler program used for the first round of nested PCR was: 95°C for 5 min; 10 cycles of [94°C for 15 sec, 75–70°C for 30 sec (0.5°C/cycle decrements), 72°C for 26 sec]; and 40 cycles of [94°C for 15 sec, 70°C for 10 sec, 72°C for 26 sec]. The thermocycler program used for the second round of nested PCR was: 95°C for 5 min; 10 cycles of [94°C for 15 sec, 68–63°C for 10 sec (0.5°C/cycle decrements), 72°C for 11 sec]; and 40 cycles of [94°C for 15 sec, 63°C for 10 sec, 72°C for 11 sec]. The final product was 167 bp.

### Myogenic differentiation of *mdx* iPSC

To induce myogenic differentiation of iPSC and ESC *in vitro*, undifferentiated cells were pre-plated on 0.1% gelatin-coated dishes for 30–45 min to remove feeder cells. These cells then underwent a modified version of the myogenic differentiation procedure described by Chang et al. [11]. Briefly, the cells spent 3 days in hanging drop culture (800 cells/20 µl), followed by 3 days in suspension culture in differentiation medium composed of DMEM, 10% FBS, 5% horse serum, 0.1 mM non-essential amino acids, and 0.1 mM 2-mercaptoethanol. The resultant embryoid bodies were then transferred to 10 cm dishes coated with Matrigel (Becton Dickinson, San Jose, CA). Medium was changed every 3–5 days. Dystrophin expression was analyzed at early (day 13) and late (day 35) stages of differentiation by both immunocytochemistry and qRT-PCR. Genes expressed in muscle progenitor cells were analyzed by qRT-PCR from samples isolated on days 6, 13, 20 and 27. Flow-cytometric (FC) analysis was carried out by staining samples from the same time course with the SM/C-2.6 monoclonal antibody (generous gift from So-ichiro Fukada). Briefly, the differentiated cells were treated with Cell Dissociation Buffer (Invitrogen) for 30 min at 37°C and gently dissociated into single cells. The cells were then labeled with either biotinylated-SM/C-2.6 (1∶100) or APC-labeled rat IgG (1∶100, as control) in separate tubes in 300 µl F10 medium with 10% horse serum (F10/10% HS) for 45 min at 4°C on a nutator. 1 ml PBS was then added and cells were centrifuged at 450 g for 5 min at 4°C. APC-labeled streptavidin was added to SM/C-2.6 treated-samples and incubated for 15 min at 4°C on a nutator. All samples were then resuspended in 200 µl F10/10% HS medium for FC analysis (on the customized “Scanford” Becton Dickinson FACScan machine, Stanford Shared FACS facility). Gene-corrected *mdx* iPSC W987, non-gene-corrected unexcised *mdx* iPSC W9 and wild-type ESC controls were differentiated and analyzed in this fashion.

### Engraftment of gene-corrected *mdx* iPSC

Animal strain maintenance, surgical procedures, and husbandry were carried out at the Veterinary Service Center of Stanford University. All procedures were approved by the Institutional Animal Care and Use Committee. Seventy-two hours before engraftment, 8 week-old *mdx/SCID* mice received 14 Gy of irradiation localized to the hind limb muscles. On the day of engraftment, SM/C-2.6-positive myogenic cells were purified by fluorescence-activated cell sorting (FACS), using a BD Aria II FACS machine and the same labeling protocol as described above for FC analysis, resuspended in 30 µl of phosphate buffered saline (PBS), loaded into an insulin syringe (BD), and injected into the left tibialis anterior (TA) muscle of anesthetized mice. 7.5×10^5^ differentiated and sorted W987 cells were injected. Control mice were injected with PBS alone. Three weeks following engraftment, TA muscles were harvested, fixed in 0.5% paraformaldehyde for 4 hours, dehydrated in 20% sucrose overnight and frozen in optimal cutting temperature (OCT) using liquid nitrogen cooled methyl-butane. Tissue blocks imbedded in OCT were cryosectioned and processed for immunocytochemical analysis using rabbit anti-dystrophin (Abcam) and rat anti-integrin (Sigma). Secondary antibodies used were donkey anti-rabbit conjugated to Alexafluor 594 and donkey anti-rat conjugated to Alexafluor 488 (Life Technologies). Nuclei were visualized using NucBlue Fixed Cell Stain (Life Technologies).

## Results

### Reprogramming plasmids and strategy

**Figure 1:**
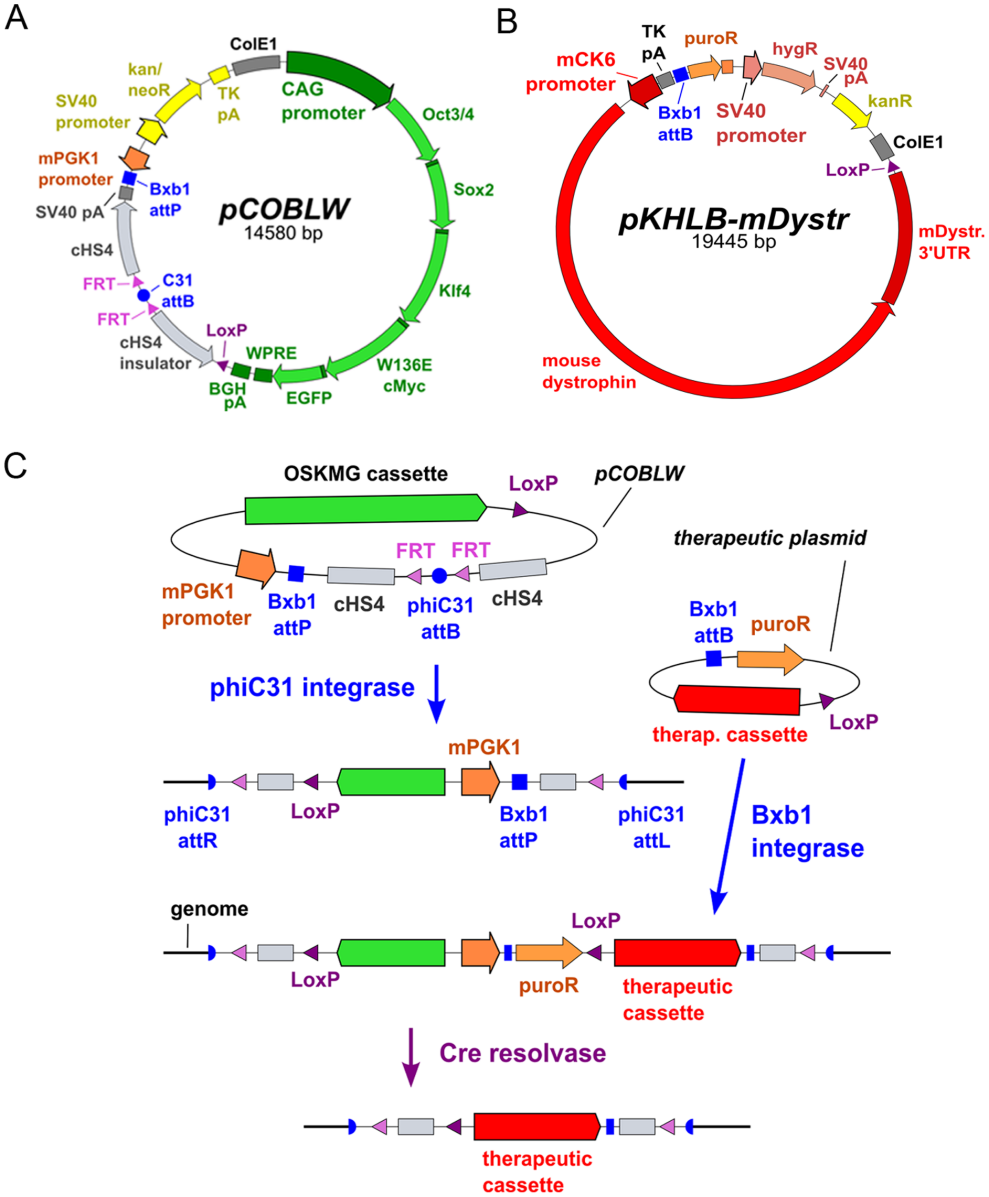
Plasmids and genome engineering strategy. (a) Reprogramming plasmid pCOBLW. (b) Therapeutic donor plasmid pKHLB-mDystr carries the full-length mouse dystrophin cDNA. (c) Schematic overview of the recombinase strategy.

To achieve reprogramming, the classic Yamanaka reprogramming genes were integrated into *mdx* adult fibroblasts by co-nucleofection of two plasmids. pVI [22] encoded phiC31 integrase [23] to mediate genomic integration at native sequences. pCOBLW (**Fig. 1a**) contained the cDNA sequences for the murine Oct3/4, Sox2, Klf4, and cMyc genes driven by the CAG promoter and connected by 2A peptide sequences to facilitate polycistronic mRNA expression. The transformation-deficient W136E version of cMyc was utilized [24]. In order to screen stable integrants easily, the EGFP reporter gene was also included near the 3′ end of the polycistronic reprogramming mRNA gene product, linked via an additional 2A peptide. The woodchuck post-transcriptional regulatory element (WPRE) was added to enhance gene expression [25]. The phiC31 *attB* site on pCOBLW permitted integrase-mediated integration of the plasmid into the genome at native pseudo *attP* sites [26], [27]. The Bxb1 *attP* site provided a landing pad for subsequent integration of the therapeutic dystrophin plasmid, pKHLB-mDystr (**Fig. 1b**), mediated by Bxb1 integrase [28]. Correct integration events connected a PGK promoter on pCOBLW with a promoterless puromycin resistance gene on the donor plasmid, allowing integrants to be easily identified by puromycin selection. Strategically located *loxP* sites were included on pCOBLW and on the therapeutic donor plasmid, so that after integration of the therapeutic plasmid, Cre resolvase [29], [30] could be used to excise the reprogramming cassette and other unnecessary sequences located between the two *loxP* sites (**Fig. 1c**). Loss of EGFP expression was used to identify excised clones. After excision, the dystrophin gene became closely flanked by insulator sequences, in order to assist expression of the transgene and reduce transcriptional effects on neighboring sequences. Only several hundred bp of foreign sequence, comprising small recombinase recognition sites, remain in the genome, flanking the insulators and dystrophin gene. Since desirable integration sites are located in intergenic regions, presence of these small residual sequences are expected to have little or no impact on safety or phenotype. FRT sites were also present in pCOBLW to permit excision of the entire plasmid with FLP resolvase, if desired.

### Generation of iPSC from *mdx* adult fibroblasts by using phiC31 integrase

**Figure 2:**
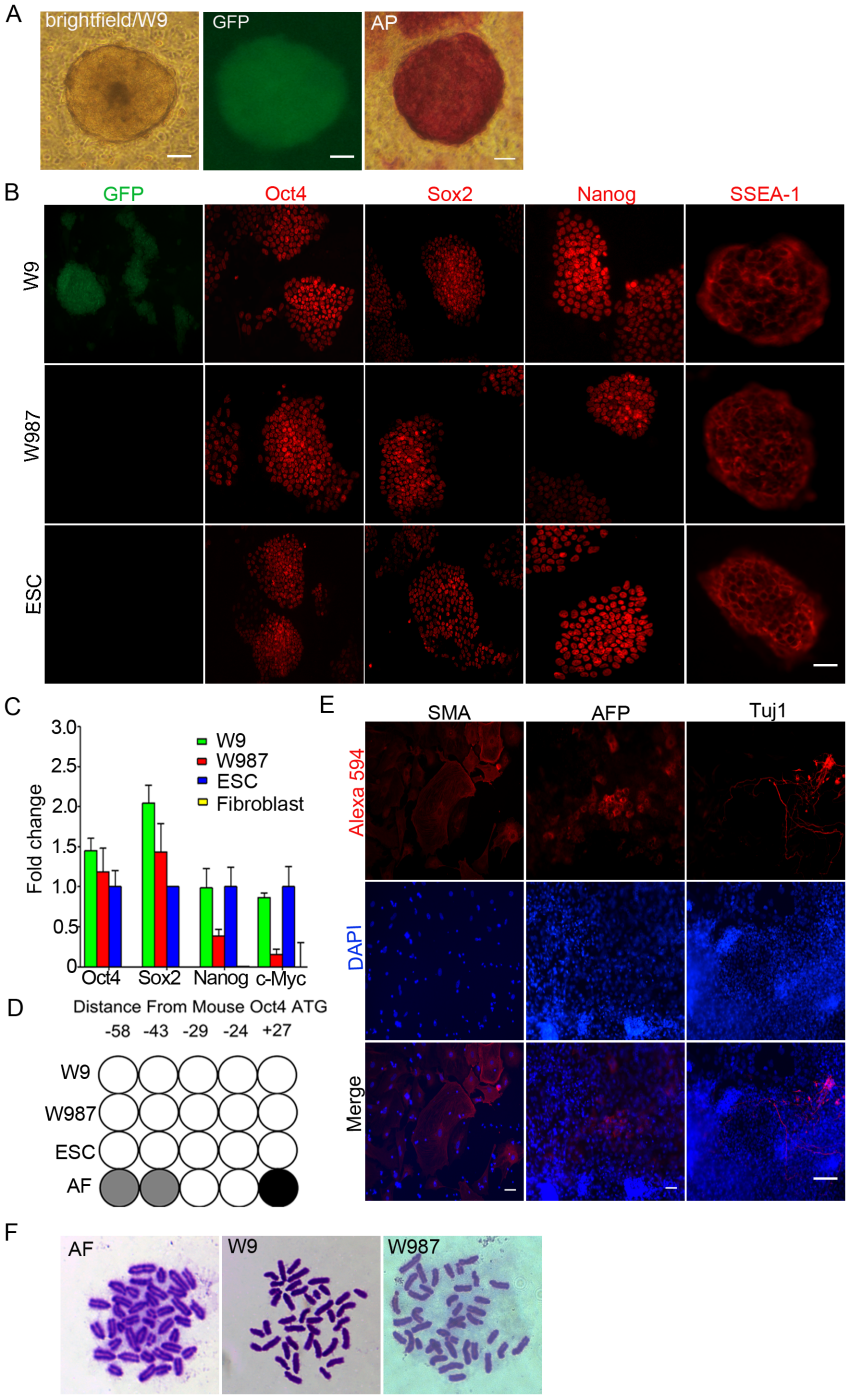
Pluripotency of *mdx* iPSC. (a) Reprogrammed *mdx* iPSC colony W9; bright field, GFP, and alkaline phosphastase staining. (b) GFP fluorescence and immunofluorescence staining of Oct4, Sox2, Nanog, and SSEA-1 in W9 iPSC before and after (W987) Cre-mediated excision of reprogramming genes and in mESC. Scale bar  = 50 µm. (c) Quantitative RT-PCR data showing expression of Oct4, Sox2, Nanog, and c-Myc in W9 and W987 iPSC, as well as in mESC controls and in the parental *mdx* adult fibroblasts. (d) Promoter methylation status of Oct4 in W9 and W987 iPSC and in mESC and adult fibroblast controls. Five different CpG islands were analyzed, indicated by their distance from the transcription start site. Open circles reflect low methylation (0–25%), gray circles represent medium (26–75%), and black circles indicate high (76–100%) methylation. (e) Embryoid bodies grown from W987 iPSC and stained for markers of the three germ layers. Day 14 embryoid bodies were stained with antibodies against smooth muscle actin (SMA), α-fetoprotein (AFP), and βIII-tubulin (Tuj1), indicating mesodermal, endodermal and ectodermal differentiation *in vitro*, respectively. DAPI was used to stain the nuclei. Alexa 594-labeled secondary antibodies were used. (f) Chromosome counts were performed in the parental *mdx* adult fibroblasts and in iPSC before (W9) and after (W987) Cre excision. The normal murine chromosome number of 40 was observed.

**Figure 3:**
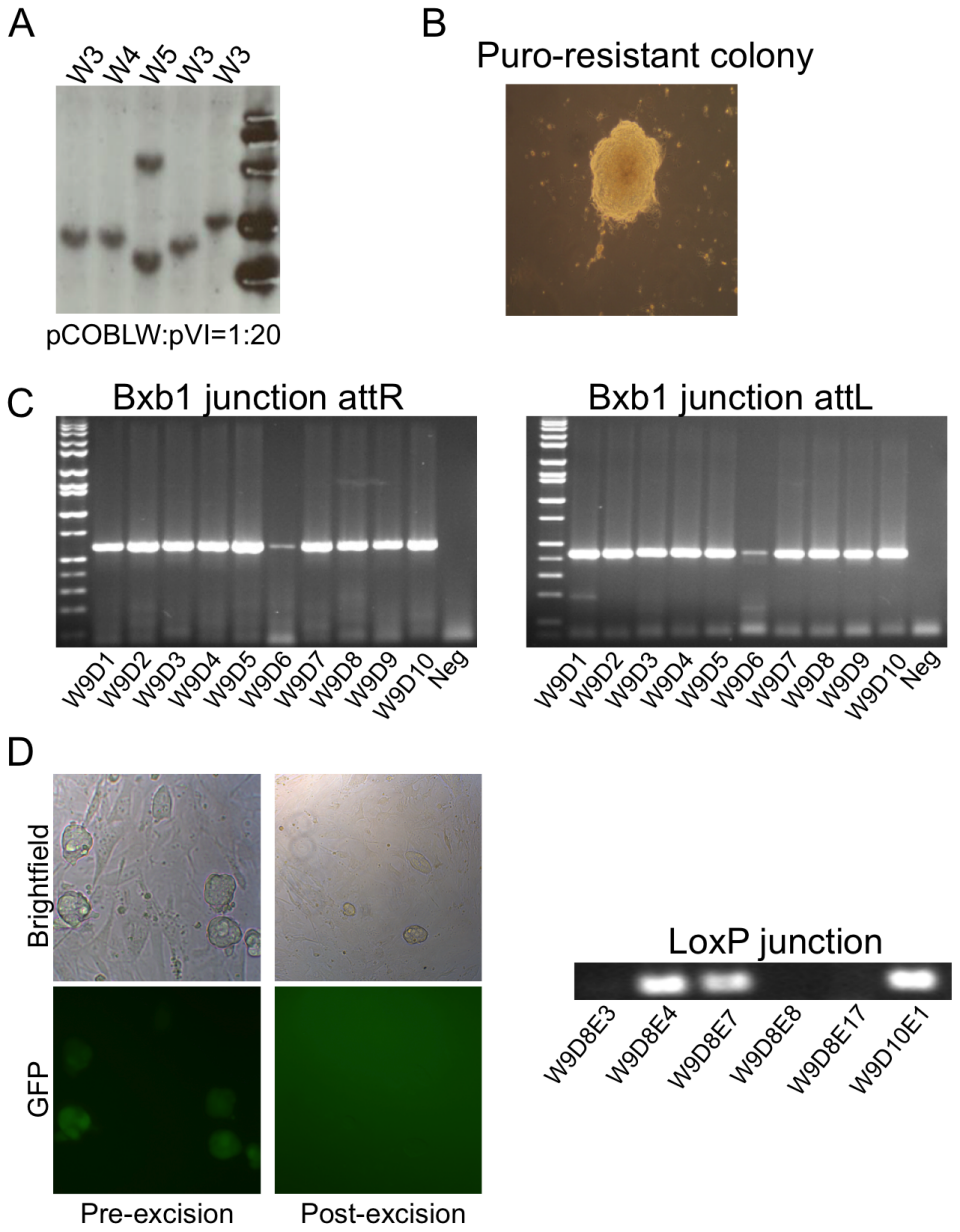
Genome engineering. (a) Southern blot analysis. iPSC clones derived from *mdx* fibroblasts and reprogrammed using pCOBLW and pVI were probed with a sequence from the EGFP gene. The number of bands indicated the number of copies of the reprogramming plasmid that integrated. W3, W4, W5, W10, and W9 represented a subset of the reprogrammed colonies that were screened. (b) Bxb1-mediated site-specific integration of therapeutic plasmid. Puromycin-resistant colonies were picked. (c) Representative subclones W9D1–W9D10 were analyzed by PCR to detect the expected *attR* (591 bp) and *attL* (431 bp) Bxb1 junction bands. (d) Left: Cre-mediated excision of unwanted sequences causes a loss of GFP fluorescence. Cre resolvase was introduced in representative subclones W9D8 and W9D10 to excise the reprogramming cassette and other sequences no longer needed. After excision, GFP expression was extinguished in colonies. Right: Representative subclones W9D8E3, W9D8E4, W9D8E7 (W987), W9D8E8, W9D8E17, and W9D10E1 were analyzed by nested PCR to detect the expected *loxP* 167 bp junction fragment indicative of successful excision.

*Mdx* adult fibroblasts were isolated and used for transfection of pCOBLW and pVI plasmids in ratios of 1∶1, 1∶7, 1∶10, and 1∶20 (w/w) to determine which ratio was optimal for obtaining reprogrammed colonies bearing a single integrated copy of pCOBLW. After co-nucleofection of the two plasmids, cells were plated in 10 cm dishes that were coated with irradiated CF1 feeder cells and transferred to mouse ESC culture medium 48 hours after nucleofection. A reprogramming efficiency of 6–10 iPSC lines per million nucleofected cells was obtained, with no difference between different plasmid ratios (data not shown). Candidate iPSC colonies were picked on day 23 after nucleofection and passaged separately. For initial evaluation of pluripotency, EGFP-positive cell lines were stained for alkaline phosphatase activity (**Fig. 2a**) and for the pluripotency markers Oct3/4, Sox2, Nanog, and SSEA-1 (**Fig. 2b**). Clones were subsequently analyzed for copy number of the reprogramming plasmid by Southern blot, probing for the EGFP reporter gene (**Figs. 3a** **and S1**). Among 16 representative *mdx* iPSC clones analyzed, derived from different plasmid ratios, 15 out of 16 (93%) exhibited a single integration event. We did not observe a significant correlation between plasmid ratio and copy number. To minimize genomic disruption, we chose to work with iPSC clones with a single integration site.

In order to evaluate the safety of the integration site, single-copy iPSC cell lines were analyzed by using ligation-mediated PCR (LM-PCR) to determine the DNA sequence of the flanking genomic DNA sequences. To reduce potential effects on chromosomal genes, we desired clones that had undergone integration into intergenic regions. The chromosomal locations of the integration sites in clones possessing a single integration in an intergenic region are shown in Figure S2. To evaluate safety, we analyzed the distances to the nearest genes, to cancer genes, and to miRNAs (**Fig. S2**) to identify “safe harbor” integration sites [31]. For the purposes of this study, we focused on clones 1∶20 #9 (W9) and 1∶10 #13 (W13), the first two single-copy, intergenic *mdx* iPSC clones we obtained that appeared to have safe integration sites, to pursue further genomic engineering. The genomic environment of the integration site in clone W9 is illustrated in **Fig. S3**.

### Addition of dystrophin cDNA to *mdx* iPSC

To correct the genetic defect in *mdx* iPSC, the W9 and W13 iPSC clones carrying one copy of the pCOBLW reprogramming plasmid were co-nucleofected with pKHLB-mDystr, encoding the full-length mouse dystrophin cDNA (**Fig. 1b**), and the pCMV-Bx plasmid encoding the Bxb1 integrase [28]. Under these conditions, the donor dystrophin plasmid became integrated at the Bxb1 *attP* site present in the integrated pCOBLW plasmid of the *mdx* iPSC (**Fig. 1c**). Integration at the correct site connected a promoterless puromycin resistance gene on the donor plasmid with a PGK promoter adjacent to the Bxb1 *attP* site on pCOBLW, permitting identification of correct integrants by puromycin selection. After 3–4 days of selection, surviving subclones were picked and expanded (**Fig. 3b**). The expected Bxb1 *attL* and *attR* junctions in 10 representative subclones from W9 (W9D1–W9D10) and 3 subclones from W13 (W13D1–W13D3) (data not shown) were verified by PCR and DNA sequencing. The PCR results indicated that 13/13 tested subclones (100%) had the expected 591 bp *attR* and 431 bp *attL* Bxb1 junction bands (**Fig. 3c**), as well as the correct DNA sequences (**Fig. S4**) indicative of precise integration of the donor plasmid at the Bxb1 *attP* site.

Once cells have been reprogrammed to a pluripotent state, the exogenous reprogramming genes are no longer needed and may be detrimental to therapeutic applications by stimulating oncogenesis and interfering with differentiation [32]. In order to delete the reprogramming cassette, two W9 subclones, W9D8 and W9D10, were transiently exposed to Cre resolvase by nucleofection with pCAG-Cre plasmid (**Fig. 1c**). Three to five days after nucleofection, colonies were examined under fluorescence microscopy, and those colonies that exhibited a loss of EGFP expression (**Fig. 3d**) were picked and expanded. The Cre-mediated removal of the reprogramming cassette from six representative subclones derived from W9D8 and W9D10 was confirmed by nested PCR and DNA sequencing. The PCR results demonstrated that 3/6 (50%) of the tested subclones exhibited the expected 167 bp *loxP* junction fragment (**Fig. 3d**) and the correct DNA sequence (**Fig. S4**) indicative of precise loss of the reprogramming cassette and associated sequences.

### Pluripotency of *mdx* iPSC before and after genetic engineering

To evaluate the pluripotency of *mdx* iPSC that were generated by using phiC31 integrase and further engineered by using Bxb1 integrase and Cre resolvase, several assays were carried out, including immunofluorescence staining of pluripotency markers, qRT-PCR analysis of the expression of genes relevant for reprogramming, spontaneous differentiation *in vitro*, and bisulfite promoter analysis. The *mdx* iPSC clone W9, having a single, safe integration site of pCOBLW, and its W987 subclone that underwent precise genomic engineering to add dystrophin and excise the reprogramming cassette, were analyzed for pluripotency, in parallel with a B6/129SvJ mouse embryonic stem cell (ESC) line used as a positive control. Both W9 and W987 had the expected nuclear staining for Oct4, Sox2 and Nanog and membrane staining for SSEA-1, and were similar in this regard to the mouse ESC (**Fig. 2b**).

Additionally, the mRNA expression profiles of the reprogramming-relevant genes Oct4, Sox2, Nanog, and c-Myc in W9 and W987 were analyzed via quantitative RT-PCR and compared with the respective transcript levels in mouse ESC, with parental *mdx* fibroblasts used as a negative control. As depicted in **Fig. 2c**, transcripts of Oct4, Sox2, Nanog, and c-Myc were detected in W9, W987, and mouse ESC and were well above transcript levels in *mdx* fibroblasts. Transcript levels of Nanog and c-Myc were higher in ESC compared to W987, which may be a consequence of the particular ESC line we used (B6/129vJ). To evaluate epigenetic changes in the DNA methylation status of the mouse Oct4 promoter region, bisulfite sequencing was carried out (**Fig. 2d**). Pyrosequencing results revealed low methylation levels in the Oct4 promoter in W9 and W987 *mdx* iPSC that were similar to the levels in mouse ESC. By contrast, the methylation levels within the Oct4 promoter region in parental *mdx* adult fibroblasts were high (**Fig. 2d**), suggesting lack of Oct4 expression, as expected.

To evaluate the potential of the fully engineered W987 iPSC to differentiate spontaneously into cells representative of the three germ layers *in vitro* as a measure of pluripotency, differentiation of iPSC subclone W987 was carried out in culture medium without LIF for 14 days. Immunofluorescence staining for smooth muscle actin (SMA), α-fetoprotein (AFP), and βIII-tubulin (Tuj1) was performed and revealed that the genetically engineered *mdx* iPSC subclone W987 retained the potential to differentiate into mesoderm, endoderm and ectoderm, further suggesting that this iPSC subclone was pluripotent (Fig. 2e). Cytogenetic analysis of parent adult fibroblasts, *mdx* iPSC clone W9, and genetically-corrected *mdx* iPSC clone W987 was carried out to determine chromosome numbers. The iPSC clones and fibroblasts exhibited the normal murine chromosome number of 40 (**Fig. 2f**), and so the iPSC were subjected to myogenic differentiation and engraftment procedures. For confirmation, similar studies were carried out on an independent iPSC clone, W12-2, derived from *mdx* embryonic fibroblasts (**Fig. S5**). The presence of the *mdx* mutation in the native dystrophin gene was verified by DNA sequencing of the appropriate region [33] in the starting mouse strain, the fully engineered W987 iPSC and iPSC line W12-2 (**Fig. S6**).

### Myogenic differentiation of genetically corrected *mdx* iPSC

**Figure 4:**
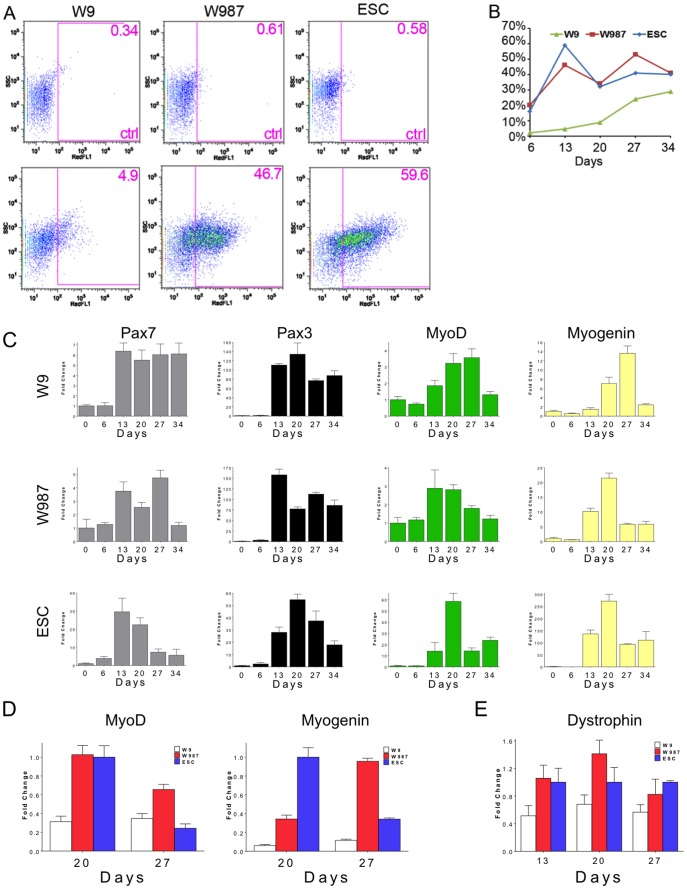
Muscle marker expression in differentiating *mdx* iPSC. W9 and W987 iPSC and ESC were differentiated *in vitro*, and at various time points, cells were stained with the SM/C-2.6 antibody and analyzed by flow cytometry (FC). (a) FC plots of W9, W987, and ESC differentiated for 13 days, indicating that approximately 4.5%, 46%, and 59% of cells were positive for SM/C-2.6, respectively, at this time point. Anti-rat IgG was used as a staining control. (b) Differentiation efficiency at the day 6, 13, 20, 27, and 34 time points for W9, W987, and ESC, as judged by percent positive for SM/C-2.6. (c) qRT-PCR analysis of muscle lineage markers *Pax7*, *Pax3*, *MyoD*, and *myogenin* during the time course in W9, W987, and ESC. (d) qRT-PCR analysis of *MyoD* and *myogenin* expression in W987, W9 and ESC cells at 20 and 27 days of differentiation. (e) qRT-PCR analysis of dystrophin expression at various time points.

To assess the potential of genetically corrected *mdx* iPSC to differentiate into muscle precursor cells, clone W987 was subjected to a myogenic differentiation protocol involving embryoid body formation and culture in horse serum [11], [19]. Differentiating W987 cells were sampled at different time points during the protocol, including days 6, 13, 20, 27, 34, and 41, and compared with the differentiation of non-corrected parent iPSC clone W9 and wild-type mouse ESC. To analyze the fraction of muscle precursor cells present in the differentiating populations, cells were mixed with monoclonal antibody SM/C-2.6, which recognizes myogenic cells and has been used to isolate quiescent mouse satellite cells [34]. FC analysis was carried out on the samples to evaluate the differentiation efficiency over the time course (**Fig. 4a**). This analysis revealed that at day 13 of differentiation, 46.7% of the W987 cells were positive for SM/C-2.6, while 59.6% of the ESC were positive at this time point. These levels were more than 9-fold higher than the 4.9% fraction of positive cells in the differentiated W9 iPSC, which lacked dystrophin expression and retained the reprogramming cassette. We speculate that continued expression of the reprogramming cassette, as documented in **Fig. S7**, may have inhibited the differentiation process in W9 [32]. In addition, it has been demonstrated that myoblasts lacking dystrophin demonstrate delayed differentiation [35]. Both W987 and ESC displayed peaks of differentiation between days 13 and day 27, gradually dropping after day 34 (**Fig. 4b**). The W9 iPSC showed a pattern of differentiation that was much less pronounced. This was reflected in the lower expression of MyoD and Myogenin in W9 cells compared to ESC and W987 cells (**Fig. 4d**). These results demonstrated that differentiation efficiency and time course of differentiation in the gene-corrected and excised W987 iPSC were roughly similar to those in mouse ESC and distinct from the parental uncorrected, unexcised *mdx* W9 iPSC.

To evaluate the expression of myogenic genes in the differentiating cultures, quantitative reverse transcription-PCR (qRT-PCR) was utilized to determine the expression levels of *Pax7*, *Pax3*, *MyoD*, and *Myogenin* (**Fig. 4c**). A surge of *Pax7* and *Pax3* expression, early markers of muscle precursor cells, occurred by day 13 in all the differentiating lines. Expression of *MyoD* and *Myogenin*, markers of more mature muscle, was skewed later in W9 compared to W987 and ESC (**Fig. 4c**). Further, expression of *MyoD* and *myogenin* were greatly reduced in W9 cells compared to W987 and ESC (**Fig. 4d**). Thus, the patterns of myogenic gene expression in W987 and ESC were similar, while expression in W9 was reduced. To assess dystrophin expression in differentiated *mdx* iPS cells before and after gene addition, qRT-PCR for dystrophin gene was carried out on samples from the differentiation time course (**Fig. 4e**). Dystrophin mRNA levels in *mdx* iPSC that experienced dystrophin gene addition were similar to levels in ESC.

**Figure 5:**
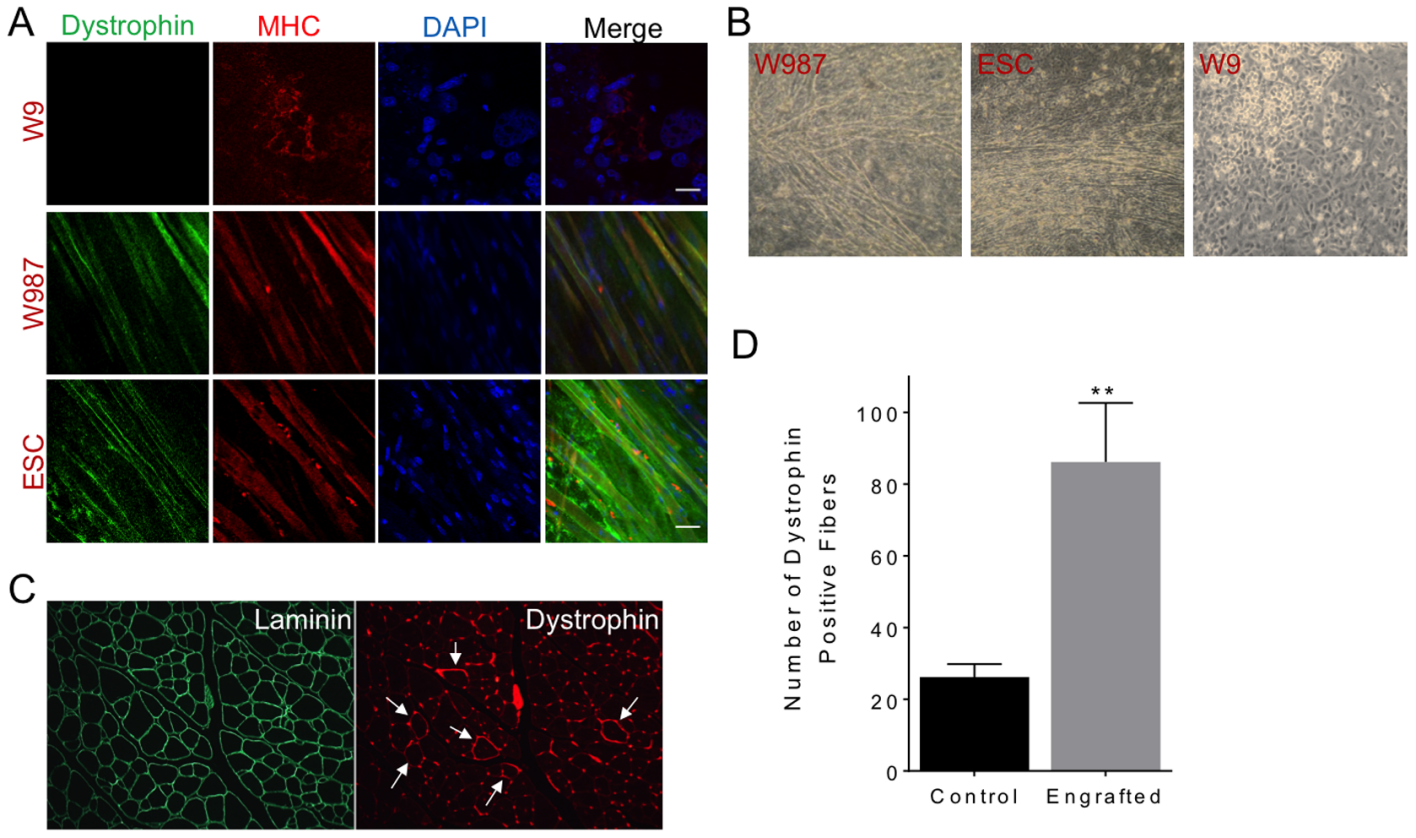
Myofiber differentiation and engraftment. (a) Immunofluorescence staining of dystrophin in W9, W987, and ESC. Myosin heavy chain (MHC) identified muscle cells after differentiation. DAPI was used to stain nuclei. (b) Myotube formation in differentiated W987 and W9 iPSC and ESC. Note that W9 iPSC did not form myotubes. (c) Engraftment of corrected *mdx* iPSC in mouse TA muscle. Approximately 750,000 W987 iPSC that were differentiated for 13 days *in vitro* and sorted for the SM/C-2.6 antibody were injected into the TA muscle of an irradiated *mdx/;SCID* mouse. After three weeks, muscle sections were prepared and stained. Staining for laminin delineated individual muscle fibers, while staining for dystrophin revealed engraftment of corrected iPSC (arrows). (d) Numbers of dystrophin-positive fibers per TA muscle, total section, are shown for engrafted muscle versus uninjected contralateral muscle (control).

To evaluate the potential of the iPSC to form myotubes *in vitro*, differentiation was carried out for at least >35 days. Myotubes formed in W987 iPSC as early as day 20 and survived for as long as 2 months, which was similar to the results in ESC, whereas myotube formation was not observed in W9 cells. Accordingly, immunofluorescence staining for dystrophin and myosin heavy chain in differentiated cultures of W987 iPSC and ESC was positive (**Fig. 5a**), which is similar to the observed increase in dystrophin RNA in engineered cells (**Fig. 4e**). W9 did not form myotubes (**Fig. 5b**), most likely because the pluripotency genes are still highly expressed compared to ESC and W987 (**Fig. S7**), since in W9 the reprogramming cassette is still present and is not expected to be silenced in integration mediated by phiC31 integrase [26]. The myotubes that formed in the W987 and ESC cultures were examined by phase contrast microscopy (**Fig. 5b**). These myotubes exhibited spontaneous, asynchronous twitching, documented in the videos available at http://www.stanford.edu/~calos/video/, further substantiating the myogenic potential of the W987 genetically-engineered iPSC.

### Engraftment of genetically engineered and differentiated *mdx* iPSC *in vivo*

To assess the engraftment potential of myogenic differentiated cells from gene-corrected *mdx* iPSC clone W987, an expanded number of W987 cells were subjected to the same differentiation protocol. Based on the robust expression of the SM/C-2.6 epitope that we observed on day 13 (**Fig. 4a & c**), our desire to engraft cells that express Pax7 (**Fig. 4c**), and that SM/C-2.6+ cells express markers commonly found on muscle precursor cells (**Fig. S8**), we chose to harvest cells on day 13 of differentiation. To enrich for differentiated cells and remove undifferentiated iPSC that might pose an oncogenic risk, candidate muscle precursor cells were purified by FACS. Accordingly, SM/C-2.6-APC positive myogenic cells were sorted by FACS. This purification resulted in a sufficient number of cells to inject three mice. From 500,000–750,000 sorted iPSC were resuspended in 30 µl of PBS and injected into tibialis anterior (TA) muscles of 8-week-old *mdx/SCID* mice that had been irradiated to enhance engraftment [36]. Three weeks following engraftment, TA muscles from the injected and contralateral legs were harvested, cryosectioned, and subjected to immunohistochemistry. Staining for laminin provided an outline of all muscle fibers, while staining for dystrophin outlined only the subset of fibers expressing dystrophin. Dystrophin fibers were detected in the injected muscles (**Fig. 5c**). To correct for the background level of dystrophin-positive fibers due to spontaneous reversion of the *mdx* mutation, which is known to accumulate progressively with age [37], the number of fibers positive for dystrophin staining was also analyzed in the contralateral, uninjected TA muscle. When the numbers of dystrophin-positive fibers were counted across the entire TA cross-sections, the numbers of fibers attributable to spontaneous reversion of the *mdx* mutation were significantly lower than the numbers of fibers detected when iPSC had been injected (**Fig. 5d**). These results provided preliminary evidence that myogenically-differentiated cells derived from *mdx* iPSC clone W987 could be engrafted in mdx/SCID mice and restored dystrophin expression in myofibers *in vivo*.

## Discussion

This study developed novel strategies to reprogram *mdx* fibroblasts and add the full-length, wild-type dystrophin coding sequence, using methods that do not involve viruses or random integration. Moreover, the work analyzed the ability of the engineered cells to differentiate and engraft in muscle. Developments in the gene therapy field have illustrated the problems that can result from random integration [38]. To move toward the type of more precise genetic engineering that may be needed for clinical application of stem cell strategies, we have taken advantage of the features of site-specific recombinases, including phiC31 integrase, Bxb1 integrase, and Cre resolvase, in a coordinated strategy (**Fig. 1c**). Each of these autonomous prokaryotic recombinases has features that are useful for genome engineering in mammalian cells.

We demonstrated the ease of reprogramming adult mouse *mdx* fibroblasts by using a plasmid system mediated by phiC31 integrase. This reprogramming system takes advantage of well-characterized features of phiC31 integrase [23], including its propensity to generate single-copy insertions in intergenic locations in unmodified mammalian cells [26], [27]. We and others have previously demonstrated the utility of phiC31 integrase for reprogramming mouse embryonic fibroblasts [39], [40], human amniotic fluid cells [40], and rat fibroblasts and adipose-derived mesenchymal stem cells [41]. This reprogramming system is simple and safe for the user, requiring a single transfection with trace amounts of non-toxic plasmid DNAs and no viral vectors, with reprogramming efficiencies comparable to those of retroviral or lentiviral vectors. Each of the iPSC clones we characterized displayed full reprogramming (**Fig. 2**). Strong expression of the classic Yamanaka reprogramming genes in pCOBLW resulted in ∼90% single-copy clones (**Figs. 3a** **, S1**) that could be easily characterized for integration site location. In this fashion, iPSC lines with desirable integration sites were identified after screening a relatively low number of clones (**Fig. S2**). The absence of packaging limits or size constraints with phage integrases made it possible to insert the sizable pCOBLW plasmid, whose design encompassed sophisticated features, including insulator sequences, transcriptional control elements, selectable markers, and recognition sites for further recombinases (**Fig. 1a**). This reprogramming system offers a viable alternative to retroviral systems, particularly for mouse cells. Newer alternatives that have been developed for human cells such as non-integrating vectors based on Epstein-Barr virus [42], [43] and mRNA reprogramming [44] are not applicable to mouse cells.

A novel application introduced here was utilization of the primary plasmid integration event to incorporate in iPSC a perfect landing pad for another site-specific phage integrase, Bxb1 integrase, which was used for integration of the therapeutic dystrophin gene. We previously characterized the activity of Bxb1 integrase in mammalian cells [28], finding that the enzyme was robust at recombining its own *attB* and *attP* recombination sites, but did not react with native sequences. These features were appropriate for the goal of inserting the dystrophin gene at a single, safe location, without creating additional integration sites. Bxb1 provided efficient integration of the donor *attB*-bearing plasmid into its pre-placed *attP* site in the mammalian genome. The specificity and precision of this reaction were approximately 100%, since every Bxb1 integrant we analyzed had recombined at the expected location, and each recombination junction sequenced was precise to the base, with no loss or gain of basepairs (**Fig. 3c** **, S4)**. As is typical for phage integrases, the absence of packaging limits or size constraints of Bxb1 integrase made it possible to readily insert the full-sized version of the lengthy dystrophin coding sequence, ensuring maximum function of the therapeutic protein. Even though the donor plasmid was nearly 20 kb in size (**Fig. 1b**), it integrated precisely into the genome at the desired position. Bxb1 integrase was thus validated as a useful tool for genome engineering strategies.

Once established, the reprogrammed state is stable; continued expression of reprogramming factors is unnecessary and can be detrimental by stimulating tumnorigenesis and inhibiting differentiation [32]. Physical excision of the reprogramming genes is effective to extinguish their expression [32], [39]. Furthermore, as a general rule in gene therapy, any DNA sequences that are not therapeutically relevant are considered an undesirable potential source of gratuitous problems, such as immunogenicity, and should be minimized. To this end, we designed the reprogramming plasmid and therapeutic donor plasmid with strategically placed *loxP* sites, so that after integration of the donor plasmid, the reprogramming cassette and most other unwanted sequences, such as plasmid backbone and selectable marker sequences, could be removed by transient exposure to Cre resolvase to mediate precise excision [30]. Cre has been widely employed in this role, as it possesses unsurpassed efficiency as a deletion agent [29].

We developed a fully engineered iPSC clone, W987, that had been reprogrammed at a safe location and in which wild-type dystrophin was inserted, with unnecessary sequences deleted. These genetic engineering steps required sequential exposure to nucleofection, recombinases, drug selection, and many passages of cell culture. It was therefore desirable to analyze whether the genetically engineered iPSC retained pluripotency, had an intact karyotype, and were capable of differentiating into myogenic precursors that could proceed to terminal differentiation and could engraft into muscle tissue. We presented evidence that pluripotency was not lost during genetic manipulation (**Fig. 2b–e**) and that karyotype was normal (**Fig. 2f**). Full pluripotency of iPSC derived with phiC31 integrase single-copy insertions of similar reprogramming plasmids has been validated by characterization of teratomas [39], [40] and chimeric mice [39].

To examine the ability of the engineered iPSC to differentiate, we adapted a differentiation protocol developed by Chang et al that had been developed for ESC [11] and extended successfully to iPSC [19]. This relatively simple protocol is based on classical formation of embryoid bodies to simulate early development [45], combined with aspects of single-fiber culture methods that had been developed for muscle, involving growth on Matrigel in the presence of horse serum [46]. Under these conditions, we observed sequential expression of typical markers of muscle development (**Fig. 4**), including dystrophin expression in the engineered cells (**Fig. 4d**, **Fig. 5a,b**) and development of mature, fused muscle fibers that contracted spontaneously in an asynchronous fashion (http://www.stanford.edu/~calos/video/). Based on our experiments it is unclear how the expression of dystrophin in our engineered cells compares to expression in ESC. If there is a difference, it could be due to features of the donor plasmid, such as the promoter or the lack of introns, or features of the integration site. We will address these potential issues in future studies in order to produce accurate dystrophin expression.

Validation that we generated functional muscle progenitor cells would be the ability of the engineered and differentiated iPSC to engraft into skeletal muscle. In an attempt to enrich for appropriately differentiated cells and to remove undifferentiated cells that could lead to teratomas, we employed sorting on a fluorescence-activated cell sorter, using the SM/C-2.6 monoclonal antibody [34]. This antibody has been shown to recognize quiescent satellite cells, which are appropriate myogenic progenitor cells for engraftment [11], [19]. The presence of dystrophin-positive fibers in muscle injected with sorted iPSC, significantly in excess of the levels of dystrophin fibers due to spontaneous reversion of the *mdx* mutation in control muscle (**Fig. 5c,d**), suggested that engraftment may have occurred. We injected cells after 13 days of differentiation, based on the expression of satellite cell markers Pax3 and Pax7 at that time point (**Fig. 4c**). However, previous studies utilized cells differentiated for 20 days [11], [19], which may be more favorable for engraftment efficiency. Use of additional markers for sorting would likely be beneficial. Effective engraftment has been shown following forced expression in iPSC of myogenic transcription factors, including Pax3, Pax7, and MyoD [13], [15]–[17], [20]. We plan to explore provision of these transcription factors using methods that avoid viral vectors and random integration.

The genetic engineering strategies developed in this study employ simple and safe methodologies for reprogramming and gene addition in mouse iPSC. These strategies could also be applied in other disorders where provision of genetically manipulated iPSC would be advantageous. We plan to continue improving genetic engineering, differentiation, and engraftment methods, as they apply to human cells.

## Supporting Information

Figure S1 **Southern blot analysis of additional** ***mdx*** **iPSC clones reprogrammed using pCOBLW and pVI.** The ratio of pCOBLW to pVI DNA used in the co-nucleofection ranged from 1∶1, 1∶7, 1∶10, and 1∶20. Representative clones from different plasmid ratios were analyzed, as indicated on the figure. These clones represented a subset of the reprogrammed colonies that were screened. 90±2% of the clones examined had one integration event. We did not observe a significant correlation between plasmid ratio and copy number.
(TIF)

Figure S2 **Integration site features of representative intergenic** ***mdx*** **iPS clones.** iPSC clones were created from *mdx* adult fibroblasts (AF) and embryonic fibroblasts (MEF). The genomic location of the integration site of pCOBLW was determined by LM-PCR. Distances to the 5′ end of the nearest gene, to known cancer genes, and to miRNA genes were analyzed as relevant features in choosing a safe integration site. We elected to focus on clone 1∶20 #9 (W9) as an *mdx* iPSC clone with an acceptable safety profile.
(TIF)

Figure S3 **Chromosome vicinity map of W9.** The iPSC clone W9 carries pCOBLW integrated at an intergenic location on chromosome 16. This chromosome vicinity map provides additional detail about the chromosomal region where pCOBLW integrated.
(TIF)

Figure S4 **DNA sequence verification of correct recombination junctions.** Upper: Analysis of Bxb1-mediated addition of the therapeutic plasmid. As shown in the schematic diagram, the *attR* and *attL* junctions that would result from Bxb1 *attB* × *attP* recombination were sequenced. The DNA sequence traces obtained upon analysis of the indicated junction regions are shown and indicate that Bxb1-mediated recombination took place that was precise to the base. Lower: As indicated in the schematic diagram, the *loxP* junction that would result from Cre-mediated excision of the reprogramming genes and other plasmid sequences was analyzed. The DNA sequence trace obtained verified that precise Cre-mediated recombination occurred.
(TIF)

Figure S5 **Characterization of** ***mdx*** **MEF-derived iPSC clone W12-2 bearing human dystrophin.** To further generalize the methodology, iPSC clones were also generated from MEF using pCOBLW and pVI. One clone with a favorable integration site,W12-2, was chosen for addition of dystrophin and further characterization. (a) Map of pKHLB_hDystrophin. This plasmid is similar to pKHLB_mDystr, but carries the full-length cDNA for human dystrophin. It was used as the donor plasmid for iPSC clone W12-2. (b) Copy number analysis. The copy number of EGFP was analyzed by TaqMan QPCR for various MEF-derived iPSC clones to determine which were single integrants. Control clone XVIII-2 (right) was a known single-integrant iPSC clone. The majority of the iPSC clones had a single copy of pCOBLW. (c) As indicated in Fig. S2, iPSC clone W12-2 was located in a safe, intergenic location. The detailed genomic location of the integration site is shown, using the UCSC Genome Browser. (d) Pluripotency immunofluorescence of W12-2 after addition of dystrophin and excision of unwanted sequences. Red coloration indicates positive staining for Oct3/4, Sox2, Nanog, and SSEA-1, respectively; blue coloration indicates DAPI. E) Embryoid body differentiation of excised W12-2 indicating the formation of all three germ layers. Red coloration denotes positive staining of respective germ layers, as described in Fig. 2; blue coloration denotes DAPI. (f) Bisulfite sequencing of CpG methylation sites in the Oct3/4 promoter indicates successful reprogramming in excised W12-2 (W12-2-hD-X). (g) Flow cytometric analysis of SM/C-2.6 staining during differentiation of excised W12-2. Cultures were analyzed on days 20 and 27. A dashed line indicates the isotype control staining profile, whereas a solid line indicates the staining profile of APC-streptavidin-bound biotin-anti-SM/C-2.6.
(TIF)

Figure S6 **DNA sequence verification of** ***mdx*** **genomic mutation.** DNA sequencing was carried out to verify that our *mdx* mice and iPSC clones derived from them were positive for the *mdx* mutation. The *mdx* mutation is a C-to-T transition at position 3185. This mutation changes a glutamine codon to a stop codon, resulting in the lack of expression of dystrophin. Chromatograms of the region of mouse dystrophin containing the *mdx* mutation were obtained by Sanger sequencing of a PCR reaction utilizing primers *mdx*F1 and *mdx*R1 [33]. The black arrow denotes the position of the mutation; the wild-type base is C, whereas the *mdx* mutation is T.
(TIF)

Figure S7 **Expression of reprogramming genes in W9, W987, and ESC.** RNA was isolated from W9 and W987 iPSC and ESC differentiated *in vitro*. RNA was harvested at 0, 6, 13, 20, and 27 days of differentiation, and qRT-PCR analysis was performed on the reprogramming factors Nanog, Oct4, and Sox2. The expression of reprogramming genes decreased after the initiation of differentiation in W987 and ESCs. W9 cells exhibited persistent expression of Nanog, Oct4, and Sox2 throughout the differentiation time course.
(TIFF)

Figure S8 **SM/C-2.6^+^ cells isolated on day 13 of differentiation express markers of satellite cells.** Plots depict flow cytometric analysis of W987 cells on day 13 of muscle differentiation. Percentages represent fractions of cells expressing the stated cell surface antigen.
(TIFF)

File S1 **Supplementary Materials and Methods.**
(RTF)
