# Supplementary figures and images for: Recombinase-Mediated Reprogramming and Dystrophin Gene Addition in mdx Mouse Induced Pluripotent Stem Cells
Source: PLoS One. 2014 Apr 29;9(4):e96279. doi: 10.1371/journal.pone.0096279 (PMC4004573; doi:10.1371/journal.pone.0096279)

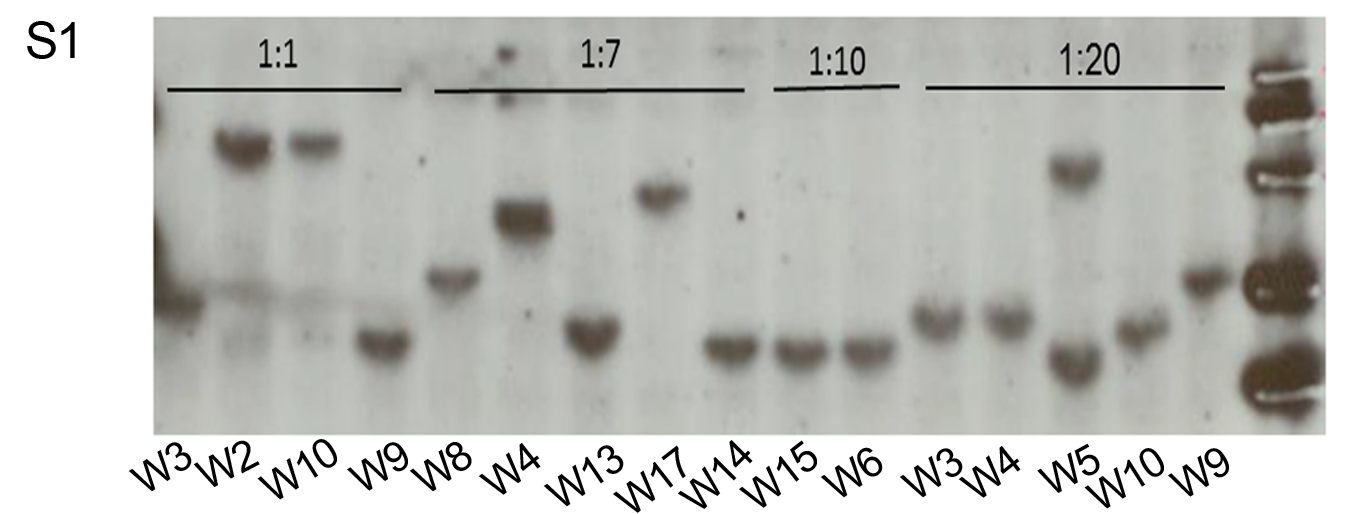

Supplement: Figure S1 — Southern blot analysis of additional mdx iPSC clones reprogrammed using pCOBLW and pVI. The ratio of pCOBLW to pVI DNA used in the co-nucleofection ranged from 1∶1, 1∶7, 1∶10, and 1∶20. Representative clones from different plasmid ratios were analyzed, as indicated on the figure. These clones represented a subset of the reprogrammed colonies that were screened. 90±2% of the clones examined had one integration event. We did not observe a significant correlation between plasmid ratio and copy number. (TIF) [file pone.0096279.s001.tif]

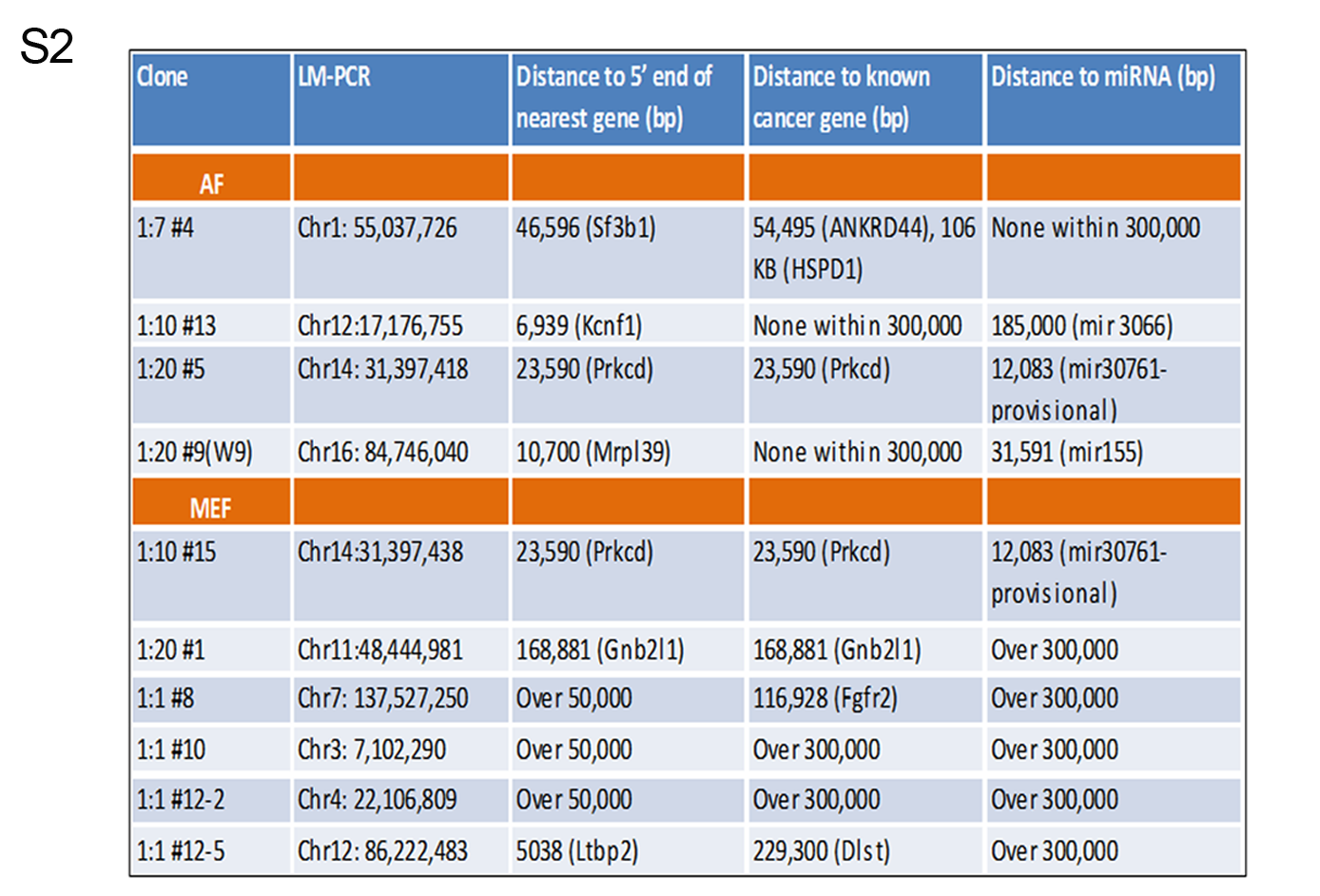

Supplement: Figure S2 — Integration site features of representative intergenic mdx iPS clones. iPSC clones were created from mdx adult fibroblasts (AF) and embryonic fibroblasts (MEF). The genomic location of the integration site of pCOBLW was determined by LM-PCR. Distances to the 5′ end of the nearest gene, to known cancer genes, and to miRNA genes were analyzed as relevant features in choosing a safe integration site. We elected to focus on clone 1∶20 #9 (W9) as an mdx iPSC clone with an acceptable safety profile. (TIF) [file pone.0096279.s002.tif]

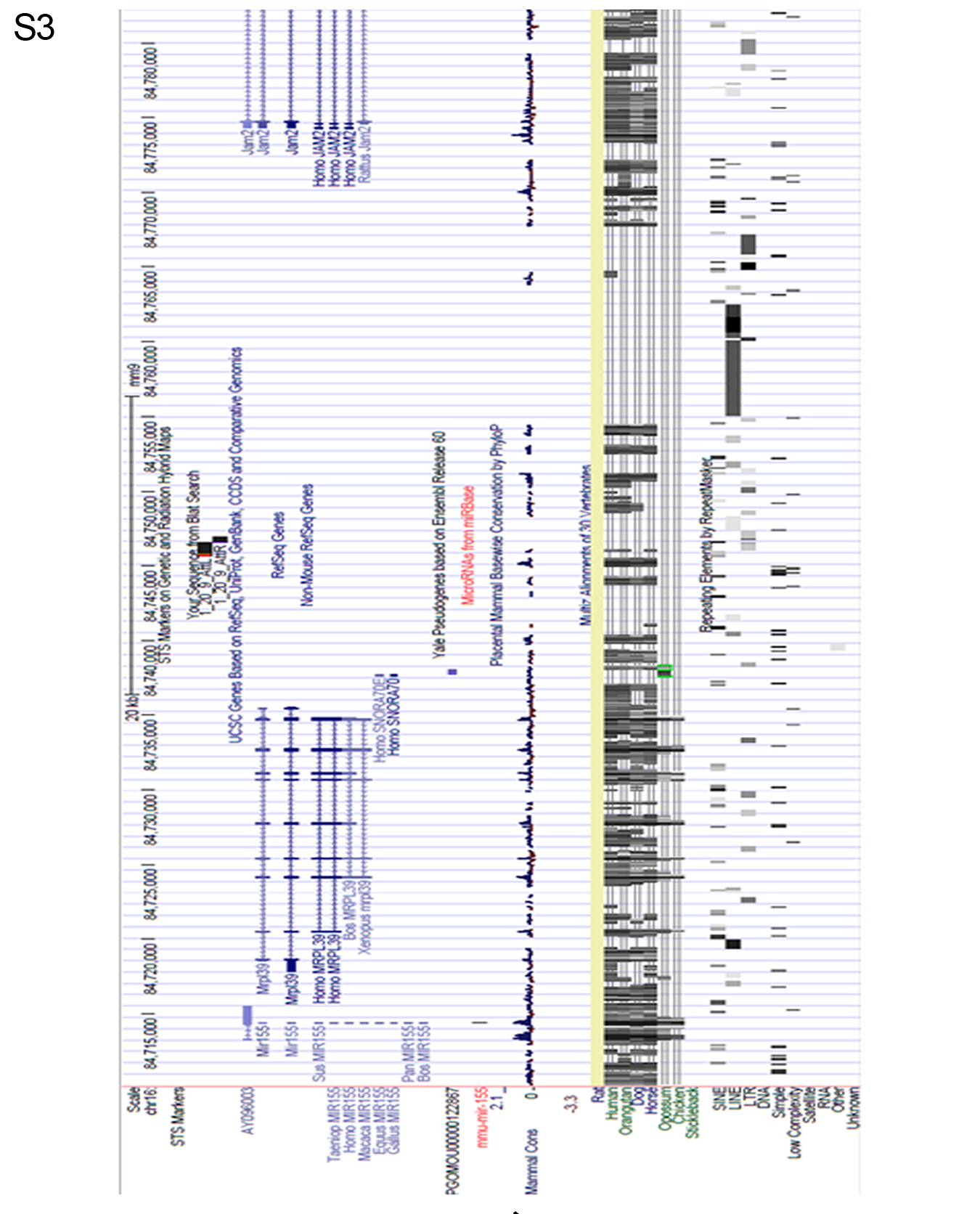

Supplement: Figure S3 — Chromosome vicinity map of W9. The iPSC clone W9 carries pCOBLW integrated at an intergenic location on chromosome 16. This chromosome vicinity map provides additional detail about the chromosomal region where pCOBLW integrated. (TIF) [file pone.0096279.s003.tif]

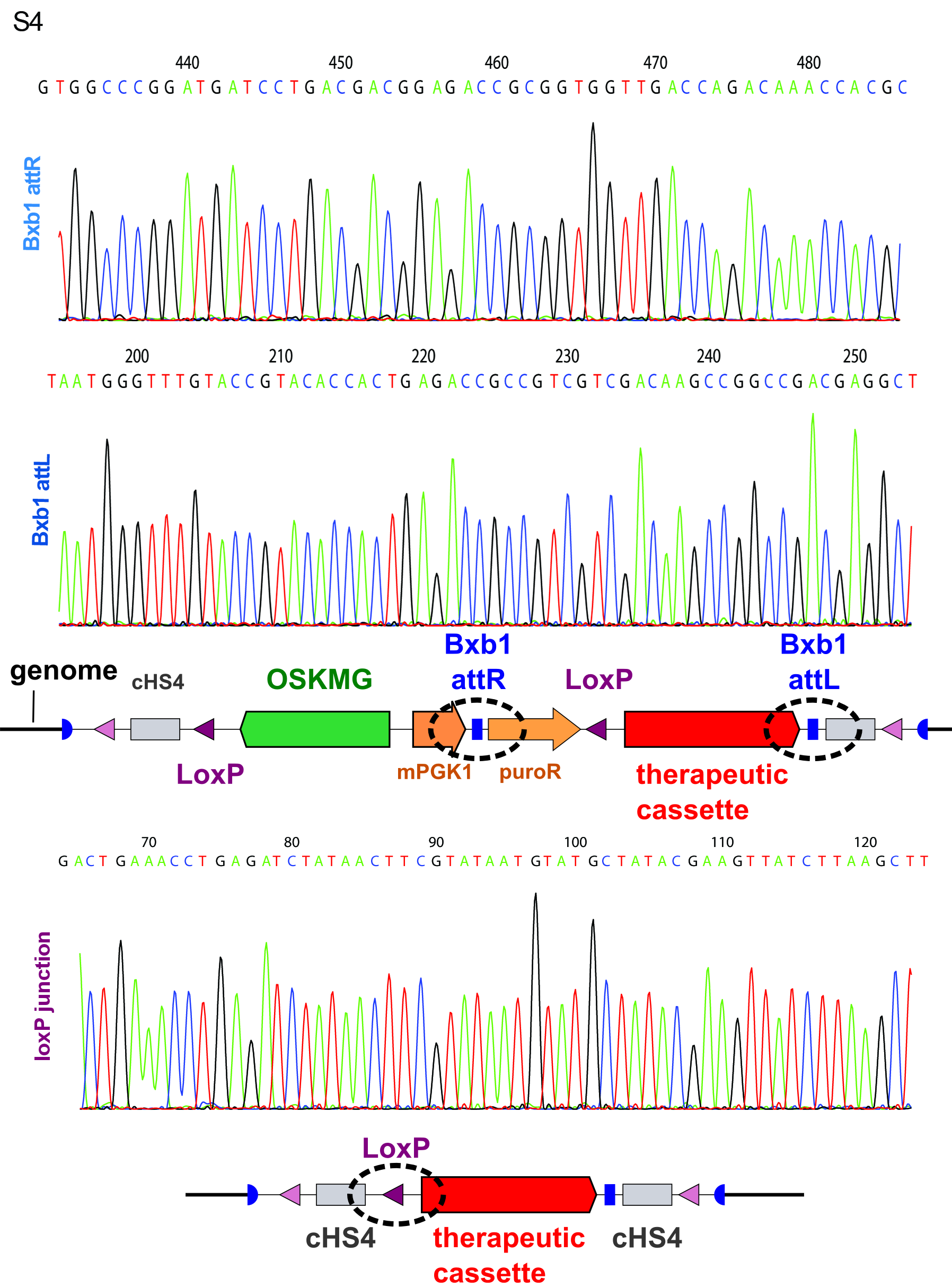

Supplement: Figure S4 — DNA sequence verification of correct recombination junctions. Upper: Analysis of Bxb1-mediated addition of the therapeutic plasmid. As shown in the schematic diagram, the attR and attL junctions that would result from Bxb1 attB × attP recombination were sequenced. The DNA sequence traces obtained upon analysis of the indicated junction regions are shown and indicate that Bxb1-mediated recombination took place that was precise to the base. Lower: As indicated in the schematic diagram, the loxP junction that would result from Cre-mediated excision of the reprogramming genes and other plasmid sequences was analyzed. The DNA sequence trace obtained verified that precise Cre-mediated recombination occurred. (TIF) [file pone.0096279.s004.tif]

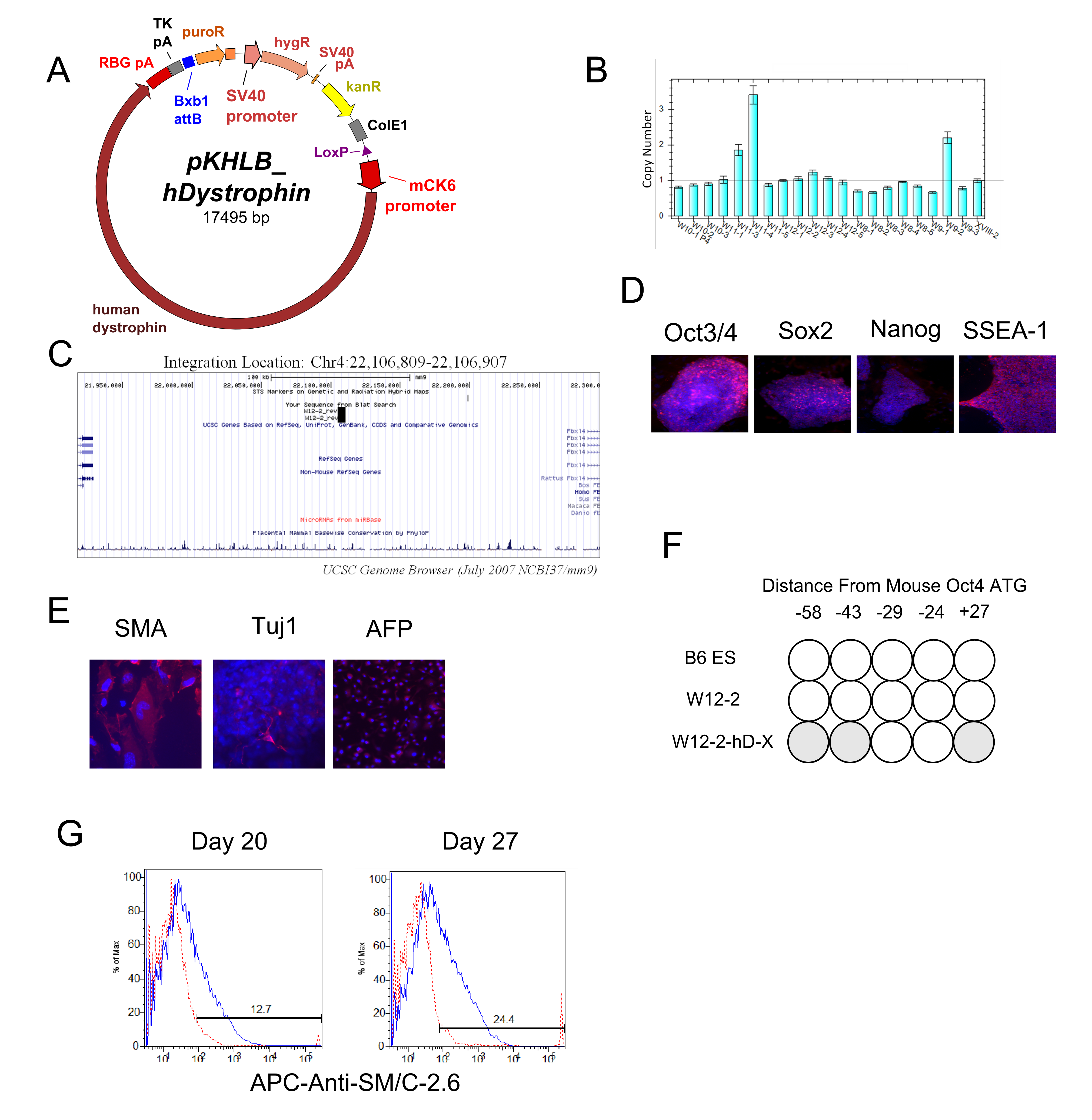

Supplement: Figure S5 — Characterization of mdx MEF-derived iPSC clone W12-2 bearing human dystrophin. To further generalize the methodology, iPSC clones were also generated from MEF using pCOBLW and pVI. One clone with a favorable integration site,W12-2, was chosen for addition of dystrophin and further characterization. (a) Map of pKHLB_hDystrophin. This plasmid is similar to pKHLB_mDystr, but carries the full-length cDNA for human dystrophin. It was used as the donor plasmid for iPSC clone W12-2. (b) Copy number analysis. The copy number of EGFP was analyzed by TaqMan QPCR for various MEF-derived iPSC clones to determine which were single integrants. Control clone XVIII-2 (right) was a known single-integrant iPSC clone. The majority of the iPSC clones had a single copy of pCOBLW. (c) As indicated in Fig. S2, iPSC clone W12-2 was located in a safe, intergenic location. The detailed genomic location of the integration site is shown, using the UCSC Genome Browser. (d) Pluripotency immunofluorescence of W12-2 after addition of dystrophin and excision of unwanted sequences. Red coloration indicates positive staining for Oct3/4, Sox2, Nanog, and SSEA-1, respectively; blue coloration indicates DAPI. E) Embryoid body differentiation of excised W12-2 indicating the formation of all three germ layers. Red coloration denotes positive staining of respective germ layers, as described in Fig. 2; blue coloration denotes DAPI. (f) Bisulfite sequencing of CpG methylation sites in the Oct3/4 promoter indicates successful reprogramming in excised W12-2 (W12-2-hD-X). (g) Flow cytometric analysis of SM/C-2.6 staining during differentiation of excised W12-2. Cultures were analyzed on days 20 and 27. A dashed line indicates the isotype control staining profile, whereas a solid line indicates the staining profile of APC-streptavidin-bound biotin-anti-SM/C-2.6. (TIF) [file pone.0096279.s005.tif]

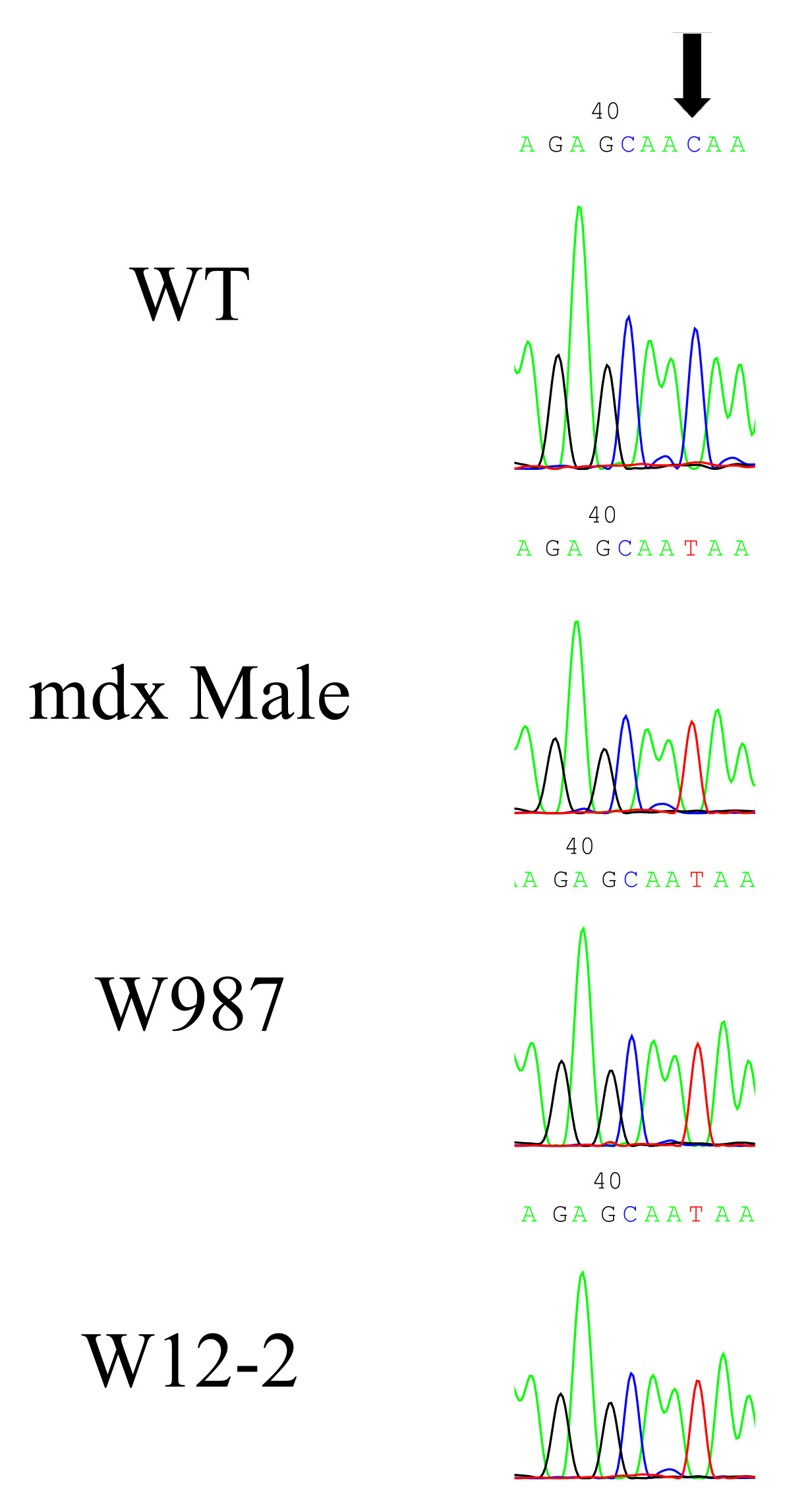

Supplement: Figure S6 — DNA sequence verification of mdx genomic mutation. DNA sequencing was carried out to verify that our mdx mice and iPSC clones derived from them were positive for the mdx mutation. The mdx mutation is a C-to-T transition at position 3185. This mutation changes a glutamine codon to a stop codon, resulting in the lack of expression of dystrophin. Chromatograms of the region of mouse dystrophin containing the mdx mutation were obtained by Sanger sequencing of a PCR reaction utilizing primers mdxF1 and mdxR1 [33]. The black arrow denotes the position of the mutation; the wild-type base is C, whereas the mdx mutation is T. (TIF) [file pone.0096279.s006.tif]

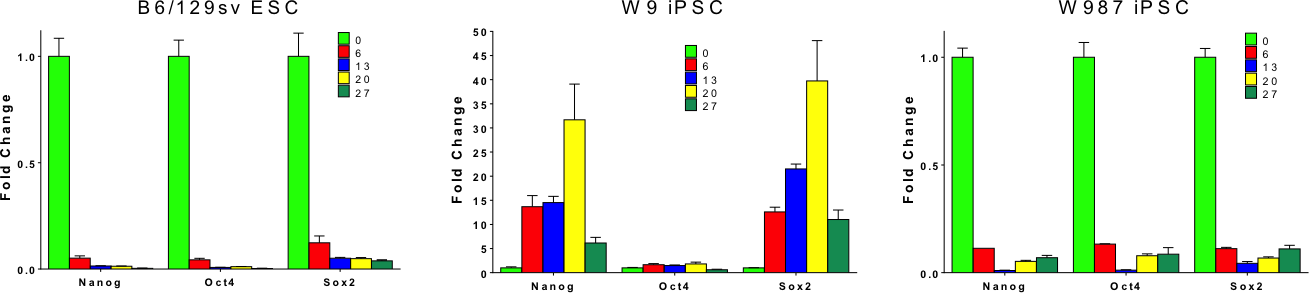

Supplement: Figure S7 — Expression of reprogramming genes in W9, W987, and ESC. RNA was isolated from W9 and W987 iPSC and ESC differentiated in vitro. RNA was harvested at 0, 6, 13, 20, and 27 days of differentiation, and qRT-PCR analysis was performed on the reprogramming factors Nanog, Oct4, and Sox2. The expression of reprogramming genes decreased after the initiation of differentiation in W987 and ESCs. W9 cells exhibited persistent expression of Nanog, Oct4, and Sox2 throughout the differentiation time course. (TIFF) [file pone.0096279.s007.tif]

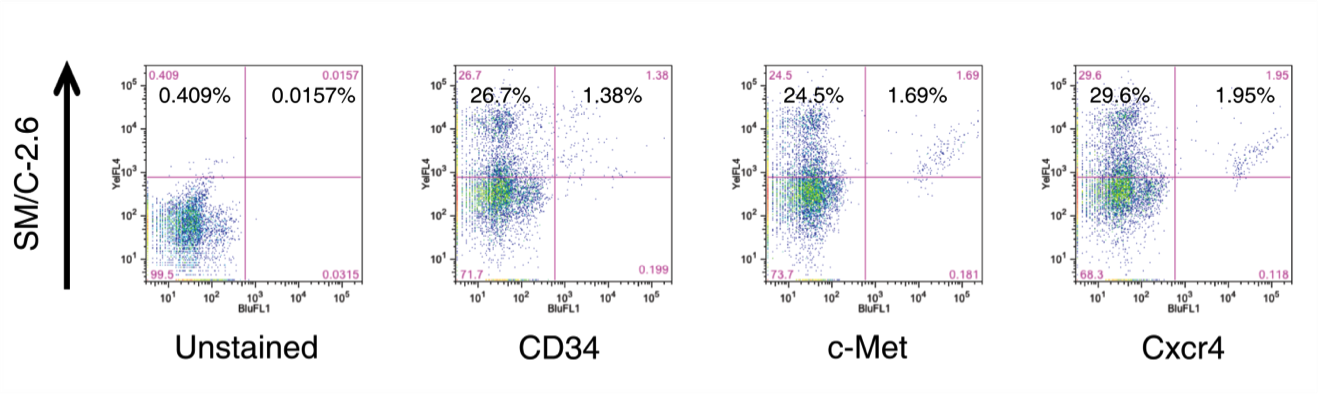

Supplement: Figure S8 — SM/C-2.6+ cells isolated on day 13 of differentiation express markers of satellite cells. Plots depict flow cytometric analysis of W987 cells on day 13 of muscle differentiation. Percentages represent fractions of cells expressing the stated cell surface antigen. (TIFF) [file pone.0096279.s008.tif]
