# Supplementary material for: Recombinase-Mediated Reprogramming and Dystrophin Gene Addition in mdx Mouse Induced Pluripotent Stem Cells
Source: PLoS One. 2014 Apr 29;9(4):e96279. doi: 10.1371/journal.pone.0096279 (PMC4004573; doi:10.1371/journal.pone.0096279)
Supplement: File S1 — Supplementary Materials and Methods. (RTF) [file pone.0096279.s009.rtf]

Supplementary Materials and Methods
Isolation of mouse fibroblasts  
	Mouse embryonic fibroblasts (MEF) were isolated from E13.5 embryos. Briefly, embryos were harvested and cut into small pieces in 0.05% trypsin-EDTA (GIBCO, Grand Island, NY) and incubated for 30 min. in a 37°C/5% CO2 incubator. Cells were then centrifuged at 300 g for 5 min and plated on 10 cm dishes coated with 0.1% gelatin (Tribioscience, Palo Alto, CA) and cultured in DMEM medium (Corning cellgro, Mediatech, Manassas, VA) with 10% FBS (Omega, Tarzana, CA).  Mdx adult fibroblasts (AF) were isolated from both skin and tail tips of 6 – 8 week old mdx male mice.  Skin and tail tips were cut into small pieces and cultured on 10 cm dishes coated with 0.1% gelatin in DMEM medium with 10% FBS until confluent, which usually took 7 – 10 days.

Construction of reprogramming and therapeutic plasmids
	The reprogramming plasmid pCOBLW (Fig. 1a) was cloned via multiple rounds of ligation between restriction-digested (RD) plasmid segments and/or RD polymerase chain reaction (PCR) products.  Phusion polymerase (New England Biolabs, Ipswich, MA) was used to generate PCR segments for cloning.  Restriction enzymes were obtained from NEB or Fermentas (Glen Burnie, MD), and every segment was gel-purified (Qiagen, Valencia, CA) prior to ligation.  The neomycin/kanamycin resistance cassette, bacterial replication origin, CMV early enhancer/chicken beta actin (CAG) promoter, 283 basepair (bp) phiC31 attB site, SV40 early 3' untranslated region (UTR), and enhanced GFP allele were all obtained from p4FLR [1].  Oct4 and the woodchuck post-transcriptional regulatory element (WPRE) were amplified from TetO-FUW-OSKM [2], while Sox2 and Klf4 were obtained from PB-TET-MKOS [3].  The transformation-deficient W136E allele of cMyc [4] was generated in vitro with PCR segments amplified from PB-TET-MKOS.  The bovine growth hormone (BGH) 3' UTR was obtained from pVax1 (Invitrogen, Carlsbad, CA), and both chicken hypersensitive site-4 (cHS4) beta-globin insulators were amplified from pJC13-1 [5].  The mouse phosphoglycerate kinase 1 (mPGK1) promoter was obtained from pPNTlox [6].  The wild-type 52 bp Bxb1 attP [7], 34 bp Cre loxP and 34 bp Flp FRT recombination sites were incorporated via synthesized oligonucleotides (Invitrogen).  The sequence of pCOBLW will be made available upon request.
	The therapeutic-template donor plasmid pKHLB-luc was cloned using the same methodology as pCOBLW.  The bacterial replication origin and kanamycin resistance cassette were amplified from pVax1 (Invitrogen), and the hygromycin resistance cassette was obtained from pSilencer 4.1-CMV hygro (Invitrogen).  The puromycin resistance ORF was amplified from pN2B-PGK-puro [8], and the luciferase expression cassette was obtained from pNBL2 [9].  The thymidine kinase and BGH 3' UTRs were amplified from pDB2 [9] and pVax1 (Invitrogen), respectively.  The wild-type 56 bp Bxb1 attB [7] and 34 bp Cre loxP recombination sites were incorporated via synthesized oligonucleotides (Invitrogen).  The therapeutic plasmid pKHLB-mDystr (Fig. 1B) was created using pKHLB-luc as a backbone.  Briefly, the CMV-luciferase cassette was removed via restriction enzyme digestion and replaced with a cassette consisting of the full-length mouse dystrophin cDNA under the control of the muscle-specific CK6 promoter (a gift from J. Chamberlain).  The DNA sequence of pKHLB-mDystr will be made available upon request.

Immunocytochemistry and alkaline phosphatase staining.
	Cells grown on 4-well glass chamber slides (Millipore, Billerica MA) were fixed with 4% (wt/vol) paraformaldehyde for 30 min, washed with 0.2% PBST, and blocked in 2% BSA in PBS plus 1% donkey serum for 1 hour at room temperature.  Cells were then incubated overnight at 4°C with primary antibodies [anti-Oct4 (Abcam, Cambridge, MA), anti-Sox2 (R&D Systems, Minneapolis, MN), anti-SSEA-1 (Santa Cruz Biotechnology, Santa Cruz, CA), anti-Nanog (Abcam)].  After washing with PBST, cells were incubated for 1 hour at room temperature with secondary antibodies labeled with Alexa 594 or Alexa 488 (Invitrogen) in 1% BSA/PBS buffer.  After washing with PBST, cells were mounted with ProLong Gold (Invitrogen).  For counterstaining of nuclei, DAPI was included in the mounting medium.
	To analyze spontaneously differentiated cells, after 10 – 14 days, cells were immunostained with antibodies against markers representing the three germ layers: smooth muscle actin (SMA, Abcam), alpha-fetoprotein (AFP, Cell Signaling Tech, Danvers, MA) and beta III–tubulin (Covance, Princeton, NJ).  Immunostaining was performed as described above.  To analyze mdx iPSC in which muscle differentiation was induced, cells were grown on 4-well chamber slides and stained with rabbit anti dystrophin polyclonal antibody (Abcam) to detect dystrophin expression in gene-corrected mdx iPS cells.  In parallel, non-gene-corrected mdx iPSC of the same line and wild-type mouse ESC were used as negative and positive controls.  The dystrophin antibody was diluted in 1% BSA/PBS at 1:200 following the immunostaining protocol described above.  Alkaline phosphatase staining was performed according to the manufacturer’s instructions (Stemgent, Cambridge, MA).  Images of stained sections were taken on an Axioshop 2 Plus microscope with an AxioCam MRc camera (Zeiss, Thornwood, NY).

Chromosome counts
	Metaphase chromosome spreads were obtained from either mdx iPSC or fibroblasts.  Cells were grown in gelatinized T25 flasks to about 70% confluency, then were treated with 0.1 μg/ml colcemid and 0.4 μg/ml ethidium bromide for 2 – 3 hours in a 37°C incubator.  Harvested cells were incubated for 20 – 30 min in 5 ml of 0.56% KCl at room temperature.  Cells were spun down at 180 – 210 g for 5 min, followed by adding 5 ml of cold fix solution (methanol:glacial acetic acid at ratio 3:1) dropwise, with flicking or vortexing, and repeated once. Dropping on slides and Giemsa staining (1:4 dilution) for 10 min were performed.  Chromosomes were counted using Image J software.  Mdx iPSC chromosome numbers were analyzed before and after Cre excision, at passages 5 and 12, respectively.  The normal mouse chromosome number is 40. 

Quantitative RT-PCR analysis of pluripotency genes
	Total RNA was prepared from mdx iPSC (unexcised and excised), mouse ESC, and mdx adult fibroblasts using TRIZOL, following the manufacturer’s instructions. 1 μg of the RNA was used for reverse transcription using the High Capacity cDNA Reverse Transcription Kit (Applied Biosystems, Carlsbad, California), following the manufacturer’s instructions.  qRT-PCR was performed using SensiFAST SYBR No-ROX kit (Bioline, Taunton, MA) and the real time PCR detection system CFX96 (Biorad, Hercules, CA).  mRNA Expression levels of Oct4, Sox2, Klf4, cMyc, Nanog, and GFP were normalized to GAPDH expression. Primers and PCR conditions were the same as in our previous study [1].

Bisulfite pyrosequencing of the Oct4 promoter
	Mdx iPS and mESC cells grown on gelatin were harvested, and genomic DNA was extracted using the DNeasy Blood and Tissue kit (Qiagen).  Primers developed by EpigenDx (Worcester, MA) were used to analyze CpG sites within the proximal promoter region of the murine Oct4 promoter.  1 μg of genomic DNA was sent to Epigendx for bisulfite treatment, PCR, and pyrosequencing.
  
In vitro spontaneous differentiation of iPSC
	For in vitro differentiation of mdx iPSC, cells were cultured on a CF1 feeder layer until 80 – 90% confluent.  Cells were pre-plated on 10 cm dishes coated with 0.1% gelatin for 30 – 60 min in a 37°C incubator, followed by embryoid body (EB) formation by suspension culture in a low attachment dish (Corning, Union City, CA).  Cells were grown in ESC culture medium in the absence of LIF.  After 4 – 6 days, EBs were transferred to 0.1% gelatin-coated dishes to differentiate for 10 – 14 days. Medium was changed every 3 – 5 days.  Cells were then immunostained following the procedure described above. 

qRT-PCR of myogenic markers
	Mdx iPSC and wild-type mouse ESC samples from the myogenic time course at day 0, 6, 13, 20, and 27 were harvested and RNA was extracted with TRIZOL following the manufacturer’s instructions.  Quantitative real-time PCR was performed using the BioRad CFX96 thermal cycler (BioRad), with Power SYBR Green qPCR Mastermix (Life Technologies, Carlsbad, CA). The following PCR conditions were used: one cycle of 50°C for 2 min and 95°C for 10 min, followed by 40 cycles of the two-step reaction 95°C (15 sec), 60°C (30 sec). Myogenic gene expression primers were as follows: Pax7 (5'-CCCTCCATGTCACCTCAAGT-3', 5'-CCAGCGGGTTTTTGTTTTTA-3'), Pax3 (5'-AACACTGGCCCTCAGTGAGTT-3', 5'-ACTCAGGATGCCATCGATGCT-3'), MyoD (5'- AGCACTACAGTGGCGACTCA-3', 5'-GCTCCACTATGCTGGACAGG-3'), and Myogenin (5'- TTGCTCAGCTCCCTCAACCAGGA-3', 5'-TGCAGATTGTGGGCGTCTGTAGG-3').  Expression levels were normalized to GAPDH (5'-TGCGACTTCAACAGCAACTC-3', 5'-ATGTAGGCCATGAGGTCCAC-3').  Amplification of dystrophin was performed using the RT2 SYBR Green Fluor qPCR Mastermix kit, using RT2 qPCR primers for dystrophin (Qiagen) according to the manufacturer’s specifications, and values were normalized to GAPDH.  Primer sequences for the reprogramming factors Nanog, Oct4 and Sox2 can be found reference 1. The following PCR conditions were used: one cycle of 95°C for 10 minutes, followed by 40 cycles of the two-step reaction 95°C (15 sec), 60°C (60 sec).


Genotyping of mdx
	Genotyping was performed by PCR using the mdxF1 and mdxR1 primers [10].  Briefly, a 179 bp product containing the mdx mutation site was amplified from 1µg of genomic DNA using Q5 High-Fidelity DNA Polymerase (New England Biolabs, Ipswich, MA), following the manufacturer’s instructions.  Genomic DNA from a female L2G85 mouse was used as a wild-type control for dystrophin, and genomic DNA from a male mdx mouse was used as a positive control for the mutation.  The following thermocycler protocol was used: 1) 98°C for 30 sec, 2) 98°C for 10 sec, 3) 53°C for 30 sec, 4) 72°C for 30 sec, 5) repeat steps 2 through 4 forty-nine times, 6) 72°C for 2 minutes.  The product was subjected to gel electrophoresis on a 1% agarose gel at 100 V before being isolated using the MinElute Gel Extraction kit (Qiagen, Valencia, CA).  The isolated product was Sanger sequenced using mdxF1 by Sequetech (Mountain View, CA).

FACS analysis
Cells were stained with biotinylated SM/C-2.6 antibody (kindly provided by Soi’ichiro Fukada) and one of the following antibodies: FITC rat anti-mouse CD34 (clone RAM34; BD Biosciences 560238), FITC rat anti-mouse CXCR4 (CD184; clone 2B11/CXCR4; BD Biosciences 551967), and anti-mouse c-Met (HGF Receptor) FITC (clone eBioclone 7; eBioscience 11-8854).  Following incubation with primary antibodies, cells were incubated with PE-Cy7 Streptavdin (BD Biosciences 557598).  All antibodies and the streptavidin were used at a dilution of 1:100.  Cells were analyzed with a FACScan Analyzer in the Stanford Shared FACS Facility using FlowJo analysis software.

Supplementary References

1.     Karow M, Chavez CL, Farruggio AP, Geisinger JM, Keravala A, et al. (2011) Site-specific recombinase strategy to create induced pluripotent stem cells efficiently with plasmid DNA. Stem Cells 29: 1696–1704. doi:10.1002/stem.730.
2.     Carey BW, Markoulaki S, Hanna J, Saha K, Gao Q, et al. (2009) Reprogramming of murine and human somatic cells using a single polycistronic vector. Proc Natl Acad Sci 106: 157–162. doi:10.1073/pnas.0811426106.
3.     Woltjen K, Michael IP, Mohseni P, Desai R, Mileikovsky M, et al. (2009) piggyBac transposition reprograms fibroblasts to induced pluripotent stem cells. Nature 458: 766–770. doi:10.1038/nature07863.
4.     Nakagawa M, Takizawa N, Narita M, Ichisaka T, Yamanaka S (2010) Promotion of direct reprogramming by transformation-deficient Myc. Proc Natl Acad Sci 107: 14152–14157. doi:10.1073/pnas.1009374107.
5.     Chung JH, Whiteley M, Felsenfeld G (1993) A 5′ element of the chicken β-globin domain serves as an insulator in human erythroid cells and protects against position effect in Drosophila. Cell 74: 505–514. doi:10.1016/0092-8674(93)80052-G.
6.     Shalaby F, Rossant J, Yamaguchi TP, Gertsenstein M, Wu X-F, et al. (1995) Failure of blood-island formation and vasculogenesis in Flk-1-deficient mice. Nature 376: 62–66. doi:10.1038/376062a0.
7.     Kim AI, Ghosh P, Aaron MA, Bibb LA, Jain S, et al. (2003) Mycobacteriophage Bxb1 integrates into the Mycobacterium smegmatis groEL1 gene. Mol Microbiol 50: 463–473. doi:10.1046/j.1365-2958.2003.03723.x.
8.     Farruggio AP, Chavez CL, Mikell CL, Calos MP (2012) Efficient reversal of phiC31 integrase recombination in mammalian cells. Biotechnol J 7: 1332–1336. doi:10.1002/biot.201200283.
9.     Keravala A, Portlock JL, Nash JA, Vitrant DG, Robbins PD, et al. (2006) PhiC31 integrase mediates integration in cultured synovial cells and enhances gene expression in rabbit joints. J Gene Med 8: 1008–1017. doi:10.1002/jgm.928.
10.   Banks GB, Combs AC, Chamberlain JS (2010) Sequencing protocols to genotype mdx, mdx4cv, and mdx5cv mice. Muscle Nerve 42: 268–270. doi:10.1002/mus.21700.
